# Integrating Biomaterials and Genome Editing Approaches to Advance Biomedical Science

**DOI:** 10.1146/annurev-bioeng-122019-121602

**Published:** 2021-04-28

**Authors:** Amr A. Abdeen, Brian D. Cosgrove, Charles A. Gersbach, Krishanu Saha

**Affiliations:** 1 Department of Biomedical Engineering, Wisconsin Institute for Discovery, University of Wisconsin–Madison, Madison, Wisconsin 53715, USA; 2 Department of Biomedical Engineering and Center for Advanced Genomic Technologies, Duke University, Durham, North Carolina 27708, USA; 3 Department of Surgery, Duke University Medical Center, Durham, North Carolina 27708, USA; 4 McPherson Eye Research Institute, Department of Pediatrics, University of Wisconsin–Madison, Madison, Wisconsin 53705, USA

**Keywords:** biomaterials, CRISPR, cell therapy, gene therapy, genome editing, epigenome editing, organoids

## Abstract

The recent discovery and subsequent development of the CRISPR–Cas9 (clustered regularly interspaced short palindromic repeat–CRISPR-associated protein 9) platform as a precise genome editing tool have transformed biomedicine. As these CRISPR-based tools have matured, multiple stages of the gene editing process and the bioengineering of human cells and tissues have advanced. Here, we highlight recent intersections in the development of biomaterials and genome editing technologies. These intersections include the delivery of macromolecules, where biomaterial platforms have been harnessed to enable nonviral delivery of genome engineering tools to cells and tissues in vivo. Further, engineering native-like biomaterial platforms for cell culture facilitates complex modeling of human development and disease when combined with genome engineering tools. Deeper integration of biomaterial platforms in these fields could play a significant role in enabling new breakthroughs in the application of gene editing for the treatment of human disease.

## 1. INTRODUCTION

**Figure 1:**
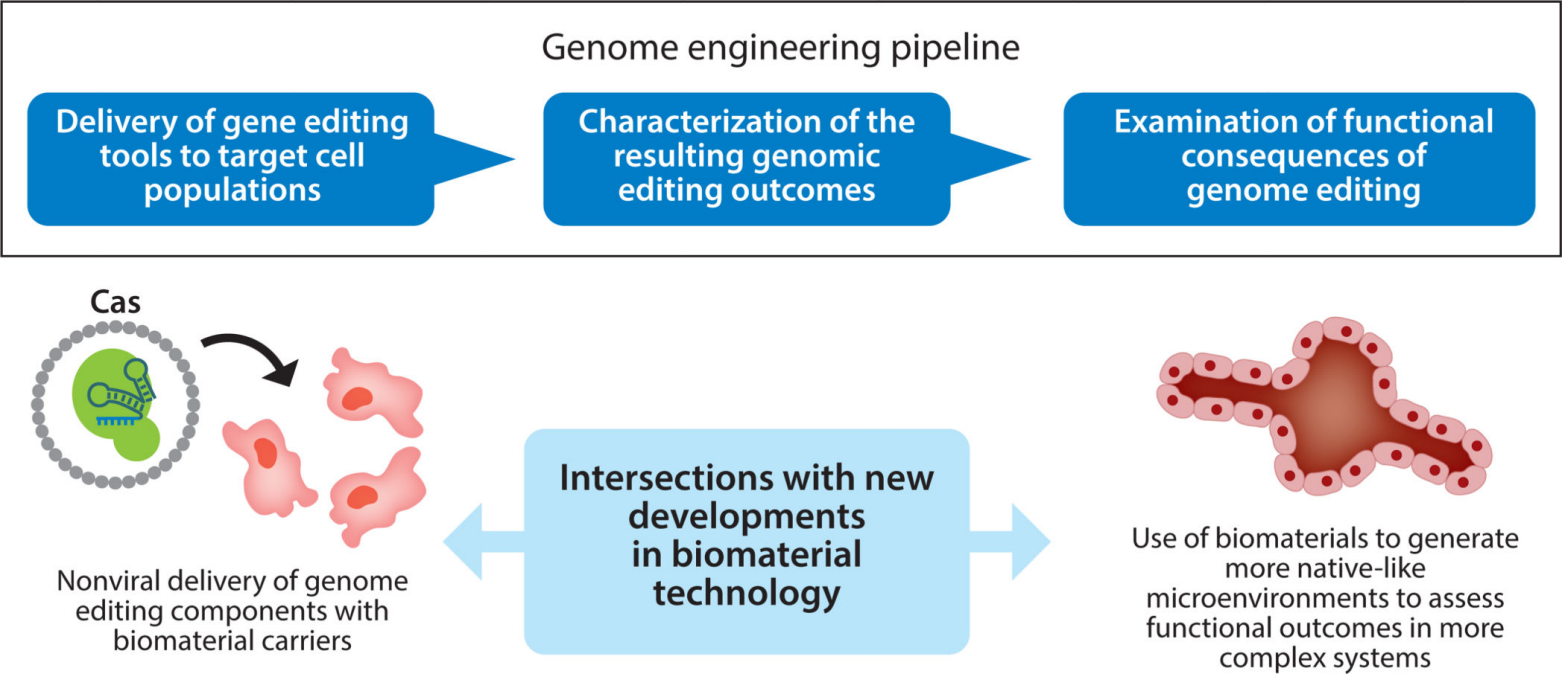
Overview of the in vitro genome editing process during the development of a gene therapy. During this process, genome editing tools are delivered to target cell populations. Next, the efficiency and types of genomic editing events are characterized. Key to the overall potency and efficacy of the gene therapy is the ability of these genomic editing events to restore the healthy function of genes affected by various mutations, and these functional improvements are typically assayed in mouse models or other in vitro model systems. Recently, the incorporation of biomaterial technologies has improved various aspects of this genome engineering workflow, including the use of biomaterials as a nonviral delivery alternative and the use of more complex biomaterials to more accurately mimic the in vivo environment when assessing the functional efficacy of gene therapy interventions.

Biomaterials have a multifaceted relationship with the genome editing field. Early developments in gene editing (1) focused on using biomaterial platforms to deliver and support genome editing tools to achieve their potential in gene and cell therapies. Concurrently, genome editing tools were introduced along with complex biomaterial culture systems to enhance the ways we study human disease and cellular behavior in more native-like contexts (2). New directions are emerging where CRISPR–Cas9 (clustered regularly interspaced short palindromic repeat–CRISPR-associated protein 9) can support the design and production of novel biomaterial platforms. In this review, we highlight how biomaterials can support the translation of somatic cell genome editors into the clinic and how biomaterial platforms can be used along with gene editing tools to model and study new developmental and disease contexts (Figure 1; see also the sidebar titled CRISPR Genome Editing Tools and the sidebar titled Genome Editing for the Design and Production of Novel Biomaterials).

## 2. BIOMATERIALS FOR THE IN VIVO DELIVERY OF GENE THERAPIES

As the number of gene therapies in clinical trials continues to increase, including both gene augmentation and genome editing, viral vectors have been the standard method for in vivo delivery. While viral delivery has been remarkably effective in several carefully selected contexts, limitations inherent to viral delivery remain a major bottleneck in the translation of some gene therapies to the clinic. For example, Luxturna, the first FDA-approved adeno-associated viral (AAV) gene therapy, treats an inherited retinal disease via delivery to the eye, which is an immune-privileged site and is amenable to local delivery of modest doses of virus. Targeting the eye bypasses some of the scale-up, cost, and safety issues that are limiting in other therapy contexts. Overcoming any of the limitations of viral vectors could greatly enhance the range of available gene therapies as well as their potency and safety.

First, biological limitations of viral vectors include the inability to directly deliver protein, the difficulty of modifying viral tropism, and the limitations in packaging size of the nucleic acid cargo inherent to each viral type. Although viral subtypes can be engineered to alter target tissue and cell type selectivity (21), it can be difficult to predict the subsequent changes in transduction and expression. Furthermore, the total dose of Cas9 delivered to each target cell is very difficult to control due to differences in the amount of DNA each cell receives as well as cell-to-cell variability in expression levels.

Second, viral vectors remain expensive to produce and scale up, and this is a major driver of the high cost of development and deployment of gene therapies (22). Quality control of viral vectors includes assaying the fraction of empty vector particles, vector potency, and impurities from the production process. Viral vectors also present unique formulation challenges, including stability at the high concentrations required for delivery, storage, and shelf life, and the cold chain from production to administration (with several of these issues becoming more apparent as product manufacturing is scaled up). Furthermore, there is batch-to-batch variability in vector preparations.

Third, there are several safety considerations with viral vectors. The viral capsid (the protein shell encapsulating viral genetic material) and viral genome can induce both innate and adaptive immune responses, and neutralizing antibodies may exist from prior natural viral infections (23, 24). Further, for some viral vectors, semirandom integration of genomic material can cause disruption of the host genome due to the uncontrolled integration process or due to the continuous expression of genome editors following payload delivery (25, 26). The difficulty of controlling the distribution of delivery of viral vectors, particularly for systemic administration, leads to delivery to untargeted tissues, which can be harmful, especially with larger doses.

There have been significant successes in addressing some of these viral delivery issues for gene therapy, as proven by the recent FDA approval and ongoing clinical trials of several viral gene therapies (27). The use of synthetic and natural biomaterials as carriers in gene therapy provides an intriguing alternative that may address many of these issues. Biomaterials have long been explored for delivery applications in gene therapy, although their adoption into clinical trials has been slow compared to viral vectors. However, recent trends indicate a renewed interest in nonviral platforms, given recent success in using nonviral platforms for vaccines and increased pressures in biomanufacturing viral vectors to meet the larger demands for gene therapy.

Biomaterial-mediated delivery approaches must overcome multiple barriers to achieve efficient cellular delivery and successful genome editing (28, 29). Particles must be stable and not self-aggregate with themselves or serum proteins upon injection. Upon entering the bloodstream, particles must avoid immune, hepatic, and renal clearance and accumulate at the desired tissue. Then, particles must be internalized by the correct cell type and release their cargo inside the cytoplasm, which often involves triggering endosomal escape (30). Throughout this process, the cargo must be protected from degradation by external host factors, such as nucleases and proteases.

**Figure 2:**
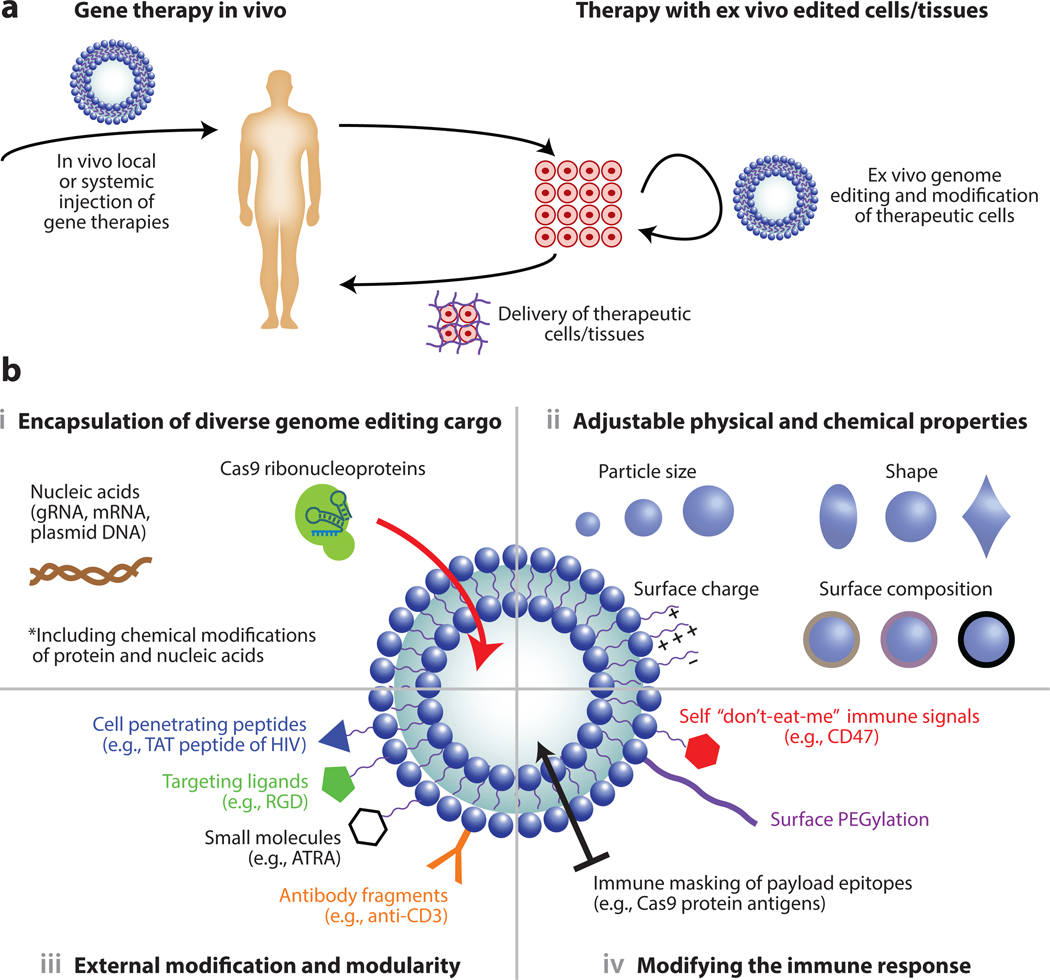
Roles for biomaterials in gene and cell therapies. (*a*) Key roles for biomaterials in gene and cell therapy include the encapsulation of genome editing payloads for systemic or local in vivo delivery and the ex vivo editing of cells/tissues to be transplanted back into the body. Biomaterials such as alginate (71) and chitosan (72) can be further used to enhance cell localization and delivery upon reimplantation into the body. (*b*) Multiple facets of biomaterial design can be leveraged to enhance delivery of diverse genome editing payloads and enable the translation of gene and cell therapies. The physical and chemical properties of particles can influence their in vivo behavior, and particle surfaces can be modified in a variety of ways. CD47 conjugation, for instance, can prevent immune cells from recognizing the biomaterial (66). Similarly, particles can be modified with cell-penetrating peptides (51), targeting ligands (49), and other moieties. Abbreviations: ATRA, all-*trans* retinoic acid; gRNA, guide RNA; HIV, human immunodeficiency virus; mRNA, messenger RNA; RGD, arginylglycylaspartic acid; TAT, transactivating transcriptional activator.

Many unique natural and synthetic biomaterials have been used for in vivo delivery. Natural biomaterials include polymers such as chitosans, lipid nanoparticles, and cell-secreted exosomes. Synthetic materials can also be polymeric [e.g., polyethylenimine, poly(β-amino esters), and poly(lactic-co-glycolic) acid], lipid based (e.g., 1,2-dioleoyl-3-trimethylammonium-propane), or inorganic (e.g., metal organic frameworks). The various barriers to gene delivery and subsequent use of various types of biomaterials for delivery have been extensively reviewed (1, 26, 28, 29, 31, 32). Here, we focus on desirable attributes of various biomaterial platforms for genome editing while contrasting some of the ways in which biomaterial properties, selection, and design can overcome some of the limitations of viral vectors in genome editing applications (Figure 2).

### 2.1. Encapsulation of Diverse Genome Editing Cargoes

CRISPR-based therapeutics have been encapsulated into various biomaterial formulations as DNA, RNA, or precomplexed RNP. Much larger nucleic acids (messenger RNA, plasmids) can be encapsulated in nanoparticles, allowing the use of larger Cas enzymes or multiple gRNAs without resorting to multiple vectors or protein splicing, as is often the case with AAV-based delivery of these components (33). The versatility of biomaterial chemistry can be compatible with a one-pot encapsulation of all of the components that may be required for gene editing, including Cas protein, gRNAs, and repair donor templates for homology directed repair (HDR) in the precise ratios required for editing (34). Furthermore, the ability to encapsulate preformulated RNPs allows the use of more efficient and stable chemically modified gRNA (35), or chemically modified Cas9 proteins that cannot be genetically encoded (36). Importantly for therapeutic approaches, the delivery of CRISPR-Cas RNPs can achieve precise temporal control of delivery of CRISPR components by titrating the number of delivered editor molecules and modulating the degradation mechanics. Such hit-and-run editing strategies lower off-target effects and increase the relative frequency of on-target editing (37).

### 2.2. Adjustable Intrinsic Physical and Chemical Properties

The physical properties of particles have a profound impact on circulation (38), clearance (39), tissue targeting, and interaction with cells (40, 41). While some delivery methodologies are limited in how much their physical properties can be modified, there are several material systems where particle size (42), shape (43, 44), and surface charge can all be robustly tailored. Furthermore, particles can be engineered to respond to stimuli such as pH, temperature, specific wavelengths of light, or specific enzyme activity. Recently, lipid-encapsulated gold nanoparticles were designed to release CRISPR-Cas9 plasmids locally at tumor sites under laser control (45). Designing particles to make use of these properties allows great flexibility in directing interactions with specific cell types across various tissues and in circulation (46). Tuning of these properties also indirectly influences the spatiotemporal delivery pattern of various gene editors (47). For example, Wu and colleagues (48) use a near-infrared-responsive nanovector that reverses its surface charge upon excitation with near-infrared light, leading to controlled release of Cas9 plasmid. Targeting PLK-1 in subcutaneous tumors, this approach was used to successfully edit tumor cells and lower tumor volume. Such an approach can lower unwanted editing spatially by only irradiating areas where editing is needed. Furthermore, by controlling when editing occurs, the amount of payload delivered can be adjusted to modulate the extent of genome editing.

### 2.3. External Modification and Modularity

In contrast to viral capsids, which require extensive protein engineering and/or high-throughput screening to modify, synthetic biomaterials can be rationally modified in a variety of different ways without affecting other functions of the delivery vehicle. For example, cell-targeting peptide ligands can be easily conjugated to the surface of polymeric nanoparticles without significantly altering their encapsulation and cellular release properties (49, 50). Conjugation can be further achieved in a modular fashion using different peptides, small molecules, or even small proteins such as nanobodies. Delivery particles are often conjugated with cell-penetrating peptides (CPPs) to enhance cellular uptake. CPPs are short and typically positively charged peptides that enhance uptake of particles when conjugated to them. One of the most common CPP sequences, transactivating transcriptional activator, was derived from human immunodeficiency virus (51). In addition, some CPPs help particles cross the blood–brain barrier (52), including delivery of CRISPR components for genome editing across the blood–brain barrier (53). CPPs have been conjugated to the Cas9 protein and gRNA to modulate cellular delivery (54), and the Cas9 protein has been directly modified with peptides or small molecules (36, 55) to aid in targeting, delivery, or stability. Other peptides can be used to control intercellular trafficking, such as the microtubule-associated sequence and nuclear localization signal, which supports transport along the cellular microtubule network for intracellular trafficking to the nucleus (56). Conjugation of small molecules to nanoparticles delivering Cas9 can modify in vivo editing outcomes. For example, all-*trans*-retinoic-acid conjugation to nanoparticles considerably increased editing upon local injection into the eye by exploiting endogenous transport of retinoids within the visual cycle (57).

With such a large design space, it can be difficult to identify optimal formulations to maximize safety and efficacy. In addition to rational design, delivery materials formed through combinatorial methods could be tested in a high-throughput fashion (58). Recently, lipid material libraries were built and large numbers of material formulations screened for delivery in vitro and in vivo through the use of next-generation sequencing of DNA barcodes (59). Using high-throughput screening, materials formulations can be identified that accumulate in different organs (59) or are functionally active (60).

### 2.4. Immune Response and Safety

Pre-existing immunity to CRISPR enzymes and viral capsids has been frequently observed in humans, presumably due to previous natural exposures to host microbes and viral infections (61, 62). This is particularly concerning for AAV-based delivery, where the pre-existing or developed immunity can limit the efficacy of the gene therapy and preclude redosing potential (63). Biomaterial platforms can protect the delivered molecules until they are inside cells, thus masking them from the immune system. However, epitopes may still be presented on the surface of cells once the cargo is released. Strategies that use biomaterials to mask vectors from the immune system include surface pegylation (64), exosomes to encapsulate gene delivery components (65), and the conjugation of self “don’t-eat-me” signals such as CD47 to the surface of nanoparticles (66). CRISPR-gold, a strategy based on DNA-coated gold nanoparticles coated with cationic polymers, was used to repeatedly dose mice for the correction of a mutation that causes muscular dystrophy without an increase in inflammatory cytokines in plasma (67). However, anti-PEG (polyethylene glycol) antibodies have also been found following nanoparticle delivery (68) and may reduce the potency of subsequent doses (69), although their full clinical effects remain unclear (64).

Another issue with most nanoparticle formulations is their accumulation in the liver following systemic delivery, which may cause unwanted editing and toxicity. Possible solutions to this drawback include improvements to targeting specificity and the design of particles that decompose without effective delivery in the liver (70).

## 3. BIOMATERIALS FOR THE DEVELOPMENT OF EX VIVO MANUFACTURED CELLULAR THERAPIES

In addition to in vivo delivery for gene therapies, biomaterials are also being developed to support the burgeoning field of ex vivo cell therapy (73). Cellular therapies make use of a variety of cell types, such as pluripotent stem cells (74), pancreatic islet cells (75), and immune effector cells (76). Autologous cells may be sourced from the patients themselves or allogeneic cells may be isolated from donors. In either case, cells need to be extracted from the body, cultured and processed ex vivo, and delivered back into the patient. Biomaterials can play a role in the culture, expansion, genome editing, quality control, and delivery of cell therapies (77). One type of cellular therapy that is actively being tested in the clinic, with FDA-approved products now in the market, is adoptive T cell immunotherapy. In this section, we expand upon the possible roles for biomaterials in editing and enhancing T cells for immunotherapy.

Cancer immunotherapies aim to activate the body’s own immune system against malignant tumors (78, 79). Chimeric antigen receptor T cell (CAR-T) therapies have become a major therapeutic strategy for several cancer targets, especially liquid tumors. In these therapies, patient T cells are collected, genetically modified ex vivo to express a tumor-targeting receptor, and then implanted back into the patient. Several CAR-T therapies for B cell malignancies have been approved by the FDA, and many clinical trials are being conducted for both solid and liquid tumors (80). Although these therapies are showing great promise, there is much room for improvement, specifically in incorporating new advances in genome editing, biomanufacturing, and delivery of therapeutic cells (81).

Typical procedures to introduce CAR-encoding transgenes for CAR-T therapies involve lentiviral vectors, which are challenging to manufacture and difficult to ensure for consistent and uniform quality. The viral production process is expensive, driving up the cost of these therapies. Moreover, the semirandom integration of CAR expression cassettes using lentiviral vectors has an associated risk of insertional mutagenesis (82). Nonviral methods have been developed recently that use electroporation and CRISPR genome editing to precisely insert CARs at known loci, with high efficiency and improved activity (83). Excitingly, this method was further improved by the use of biomaterials as adjuvants for gene transfer to enhance delivery of genome editors in vitro (84, 85). The development of efficient methods for biomaterial-mediated introduction of CARs into T cells can significantly improve the process and overcome the issues with using viral transduction and the low throughput of electroporation. Additionally, biomanufacturing of CAR-T cells involves harvest, expansion, and activation of T cells, alongside additional quality control. Multiple groups have taken advantage of the defined physical, structural, and chemical properties of biomaterials to control the T cell culture process (71). Improving the survival and potency of T cells leads to more potent therapies and lowers required cell numbers, a major bottleneck in biomanufacturing. Using soft silicone substrates for T cell culture leads to improved T cell activation and proliferation (86). Biomaterial-based artificial antigen-presenting cells have been used to activate specific subsets of T cells with controlled densities of multivalent cues (87, 88) or immobilized cytokines (71, 87). In addition to activation, biomaterials can provide structured synthetic scaffolds to expand and monitor T cells (88) and assay their potency (89).

To enhance T cell therapies in vivo, nanoparticles have been attached to T cells to co-deliver immune-modulating factors (90, 91). Multiple materials have also been developed for the delivery and localization of T cells in vivo. Scaffolds can be used to localize T cells to desired sites and co-deliver stimulatory molecules (92). For example, alginate scaffolds that present stimulatory and migratory signals to T cells improved their proliferation and activity at tumor sites (93). Similarly, implanted chitosan gels have been shown to support T cell expansion and phenotype in vivo (72). Micropatterned conforming nitinol thin films have been used to locally deliver T cells to tumors at high density in solid tumors (94). Finally, targeting T cells in vivo for genome editing by the delivery of nanoparticles designed to specifically target and edit T cells may eliminate laborious and expensive ex vivo processing altogether (95).

These studies demonstrate the importance of the culture environment and its influence on cellular behavior. Since most studies are performed in vitro, cells are usually studied in a context that is very different from application. As biomaterials and genome editing tools develop, scientists are able to generate more sophisticated native-like environments in vitro to study and control the behavior of cells, leading to increasingly more accurate microphysiological and disease models.

## 4. BIOMATERIAL AND GENOME ENGINEERING INTERFACES TO STUDY HUMAN DISEASE

While most genome editing work takes place in cells cultured in rigid 2D culture dishes, recent studies have begun to explore how engineered native-like cellular niches can recapitulate in vivo behaviors without the complexity of in vivo model systems. These systems can be exceedingly simple, such as embedding cells in 3D agar or laminin as an assay for cancer cell growth (96–98), or they can be highly complex systems that include logic-gated synthetic materials that can respond or be remodeled in a cell-mediated fashion (99–102). Key to the ability of any of these systems to more accurately mimic the native microenvironment is their provision of the proper cell–cell communication and organization, material stiffness, nutrient and oxygen diffusion, and cellular polarity that is present in the native extracellular milieu (103). While various forms of simple 3D culture systems have been in use for many decades, recently these systems have exploded in both popularity and complexity. The use and development of these 3D culture platforms to support complex cell cultures have recently been coined as organoid engineering (104).

While several stem cell types can be expanded in optimized culture conditions on rigid 2D tissue culture dishes, there are numerous cell types relevant to tissue development, repair, and disease progression that cannot readily be grown in the absence of a more complex environment. One of the first examples of utilizing an organoid culture system to maintain these stem cell populations explored using 3D Matrigel encapsulation combined with R-spondin1 ligand presentation to maintain Lgr5^+^ intestinal stem cells in vitro (105). Surprisingly, after only 14 days of culture in this system, these initial conditions allowed cells to form 3D structures with crypt-like buds, wherein different subpopulations of stem cells exhibited different Wnt signaling responsiveness. These gut organoids could be created using cells isolated from embryonic to adult tissue, and as a result could model a variety of developmental or pathogenic stages in this digestive tissue system. Since this early example, organoid cultures have been established for a variety of tissue systems, including the optic cup (106), neuronal/brain structures (107–109), the liver (110), a variety of cancers (111), and other tissues (112).

The use of Matrigel as the biomaterial substrate has been a constant component of nearly all the aforementioned organoid systems. Matrigel is a decellularized basement membrane formulation produced from Engelbreth–Holm–Swarm mouse sarcoma tumors that includes growth factors and other proteins from this niche. While the use of Matrigel has been highly supportive of the culture of many different complex structures and cell types, it has many drawbacks that have begun to limit its usability. These drawbacks include the fact that Matrigel is derived from a tumorigenic niche, has poorly defined levels of constituent proteins, has limited capacity for engineered tunability, can be prohibitively expensive, and exhibits significant lot-to-lot variability (113–115), which limits both the scalability and the complexity of the environments that can be modeled with Matrigel.

Recent developments in the biomaterial field have led to the creation of synthetic biomaterial platforms to replace Matrigel in a variety of use cases (115). These synthetic materials are highly scalable, have precisely tuned mechanics, and can be engineered for precise spatiotemporal presentation of key cues to cells. Recent work has utilized these systems to allow for proper stem cell maintenance and behavior (102, 116), as well as to engender more precise control of organoid development (117). For example, a well-defined synthetic biomaterial niche for intestinal stem cell expansion was developed that consists of a stiff (elastic modulus *E* = 1.3 kPa) proteolytically degradable 3D PEG hydrogel system expressing the RGD peptide from fibronectin, followed by organoid formation and culture in extremely soft environments (elastic modulus *E* = 190 Pa) with laminin-111 presentation (117). Continued progress in biomaterial formulations for organoid development will include advances in time-dependent material properties (118–123), microtissue assembly and architecture, and complex multiorganoid interactions (124–126) that will allow for the in vitro analysis of gene editing in diverse cell types and cellular niches.

### 4.1. Gene Editing to Model Human Disease

**Figure 3:**
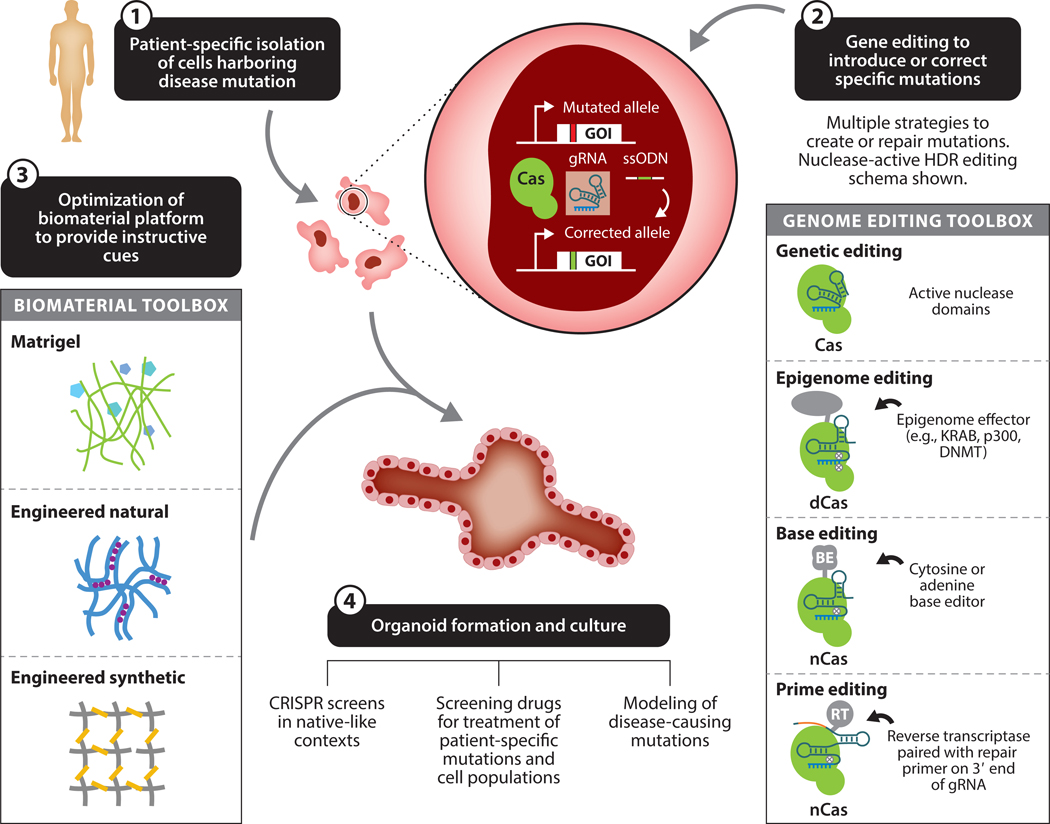
Overview of how biomaterials and gene editing tools are being combined to study human disease in a native-like biomaterial context. (➊) The power of this approach is that patient-specific cells can be isolated and used to study the disease-causing mutation in the same genetic background as the patient. Either before creation of an organoid, or after the cells are incorporated into an organoid, genome engineering tools (➋) can be introduced to correct or introduce mutations into the patient’s cells. Shown is a typical approach for nuclease-active Cas9 using HDR. In this approach, a mutated allele in the gene of interest is targeted using Cas9 protein, a gRNA, and an ssODN, allowing for precise repair of the mutation. Other approaches can be used to introduce precise genome edits besides nuclease-active Cas9, which include the introduction of epigenome-modifying effector domains fused to nuclease-deactivated dCas9 for precise epigenome editing and gene regulation, BEs that use an nCas9 (where one nuclease domain is deactivated) fused to an enzyme such as APOBEC1 that can catalyze a single DNA base change, and prime editing where a reverse transcriptase is tethered to the nCas9 protein and can subsequently direct repair from a template on the 3′ end of the gRNA. Following any of these genome engineering functions, these cells can be combined with several biomaterial platforms (➌) that allow for complex 3D cultures of cell populations that would otherwise not readily be maintained on standard tissue culture plastic. Traditionally, Matrigel has been used to support the formation of these cultures, but engineered natural hydrogels (e.g., agar, alginate, methylcellulose, hyaluronic acid) and engineered synthetic hydrogel systems (e.g., polyethylene glycol, polyacrylamide) have independently tunable characteristics including stiffness, degradability, and ligand presentation that can further enhance these systems. Following the generation of these organoids from patient-specific cells and the desired genome engineering procedure (➍), these more native-like environments can be used to provide new insights into treatments for patient-specific disease mutations. Abbreviations: BE, base editor; dCas9, nuclease-deactivated Cas9; DNMT, DNA methyltransferase; GOI, gene of interest; gRNA, guide RNA; HDR, homology directed repair; KRAB, Krüppel-associated box; nCas9, nickase-Cas9; RT, reverse transcriptase; ssODN, single-stranded oligonucleotide donor.

Numerous diseases involve mutations in genes that are only expressed in certain cell types or arise from de novo mutations that manifest as pathogenic only in certain cellular subpopulations within the tissue (127). Therefore, testing different genome engineering strategies to correct different mutations in relevant cell types requires the appropriate in vitro culture systems (128, 129) (Figure 3). One clear advantage of the use of organoids or other 3D systems for studying gene editing is that many of these organoid cultures can be derived from progenitor cells directly isolated from diseased patients or from patient-derived induced pluripotent stem cell (iPSC) lines (130, 131). Mutations that lead to disease phenotypes can often be widely variant between patients, requiring patient-specific modeling to accurately assess potential gene editing strategies in correcting the disease-causing mutation. For example, while there are hot spots of Duchenne muscular dystrophy mutations from exon 45–55, there are several thousand unique mutations that can lead to Duchenne muscular dystrophy, and these mutations span nearly the whole dystrophin gene (132, 133). Patient-derived organoid systems have the advantage of being able to directly test gene editing strategies for various mutations across an isogenic donor background in both healthy and diseased contexts. In combination with these patient-derived cells, gene editing tools can be used to either correct the specific mutation that gave rise to a diseased phenotype or even introduce new pathogenic mutations in otherwise healthy cells to model and study patient-specific disease responses.

One of the first such studies utilizing CRISPR-Cas9 editing in conjunction with organoid systems was performed using an intestinal stem cell organoid culture platform to make corrections in cystic fibrosis by targeting the mutated *CFTR* gene. Schwank and colleagues (134) used human intestinal stem cells to create organoids and further used the CRISPR-Cas9 system to correct the homozygous F508 mutation in *CFTR* via HDR to allow for proper CFTR protein folding and processing. Following this genomic editing, the authors highlighted improved functional responses of the patient-derived organoids to a forskolin swelling assay that challenged the function of *CFTR*. This example was the first instance of a successful therapeutic intervention using CRISPR-Cas9 editing in human-derived tissue and began to illuminate the potential for organoid systems to model both efficacy of editing and any unexpected or off-target editing outcomes in human cells.

Subsequently, there have been many more examples of how organoids enable the study of genome editing of human disease in the context of patient-specific mutations. Some of the first work to model human disease progression using CRISPR-Cas9 systems focused on early biologic mutations that can lead to cancer progression. One such approach relied on knocking out key DNA repair genes *MLH1* and *NTHL1* to mimic early mutations in the DNA repair machinery that can lead to cancer development (135), whereas other work utilized organoids harboring five simultaneous mutations in tumor suppressors and oncogenes (*APC*, *SMAD4*, *TP53*, *KRAS*, and *PIK3CA*) to model the complex mutational landscape that can arise in cancer (136). While these examples highlight cases where a set of known mutations can lead to disease progression, disease states in which there is no good genetic model for the disease have also been addressed with organoid culture systems. The use of iPSC-derived brain organoids led to the discovery of FOXG1 causing overproduction of GABAergic neurons in autism spectrum disorder (137). Additionally, through collecting many paired biopsies of tumor samples and healthy control tissue from patients with colorectal cancer, researchers have created an organoid biobank where they can more systematically examine an entire landscape of mutations that can drive colorectal cancer development through unique means (138). Besides these detailed examples, organoids have been used in conjunction with CRISPR-Cas9 to model numerous facets of human disease (139, 140).

### 4.2. High-Throughput Screening with CRISPR Systems

Recently, the CRISPR-Cas9 platform has emerged as a remarkably powerful and adaptable tool for use in genomic screening approaches to interrogate genetic interactions in an unbiased fashion. In these approaches, cell populations are typically transduced with virus to express Cas9 combined with a library of assorted gRNA sequences, such that each cell receives only a single gRNA and therefore a unique genetic perturbation. Following transduction, cells are then enriched based on a selectable phenotype of interest (such as cell growth, reporter gene expression, and drug resistance), and over-represented or depleted gRNAs in these selected populations are determined via next-generation DNA sequencing. The first wave of these screens was performed on cells in suspension culture or on tissue culture plastic dishes (141–151), and many CRISPR screens are still performed in these conditions due to the high cell numbers required to maintain the proper coverage of large libraries. While clearly there is much to be learned from these screens using cells on standard culture conditions, these experimental setups cannot robustly recapitulate many features that arise in the native cellular niche, including complex interactions between multiple cell types, nutrient usage and metabolism, biophysical context and architecture that arise from cell–extracellular matrix contact, and other features present in the native microenvironmental milieu. To address these concerns, in vivo screens have been performed with CRISPR systems that allow researchers to examine and perturb more complex gene network interactions in their native context (152–160). However, these in vivo screens have a significant number of technical challenges that prevent their widespread usage. Though these screens can be performed with human cells in xenograft mouse models, niches from other species do not fully represent the human in vivo context. Additionally, many tissue niches are not amenable to screening due to limits on proliferation, cell number, and delivery, and as such in vivo screens to date have mostly focused on cancer model systems (161).

As a compromise between the simplicity of 2D screens and the physiological relevance of in vivo CRISPR screens, more complex 3D material systems can be combined with CRISPR-Cas9 screening techniques to generate important new insights into disease progression. Recent work on this front has elegantly shown how small shifts in the cellular biomaterial niche can lead to large changes in high-throughput CRISPR screening results. For example, many tumor-suppressor genes do not exhibit positive growth effects when knocked out in 2D monolayer (162). To examine this discrepancy further, a genome-wide Cas9 nuclease screen was performed using NCI-H23 lung cancer cell lines in both 2D monolayers and 3D methylcellulose spheroids. Through this work, the knockout of tumor suppressors in 3D culture conditions was confirmed to have a positive growth effect, leading to identification of thousands of hits that were only identified in 3D culture and corresponding paired in vivo screens. Especially interesting was that these gene hits found in the 3D environment but not in 2D monolayers were more enriched for mutations in lung cancer. Other recent work has leveraged intestinal organoid systems along with CRISPR screening to examine how cancer cells develop resistance to transforming growth factor β and overcome its tumor-suppressive capability (2, 163). These examples highlight the importance of using biomaterial platforms capable of mimicking the native microenvironment to find biologically meaningful disease interventions.

Organoids and other complex 3D culture environments are also excellent test beds for high-throughput screening approaches to see how various drugs can combine with patient-specific mutations to ameliorate disease phenotypes. To accomplish this requires organoid formation and culture in a highly reproducible and controllable fashion in order to accomplish drug screening at an industrial scale. Accordingly, recent work in the biomaterials field has focused optimized parameters for the high-throughput generation, culture, and downstream phenotypic analysis of human PSC–derived organoids to screen drugs (164–169). Typical to these approaches is the use of Matrigel and polydimethylsiloxane as culture substrates, but one recent system developed U-shaped microcavities cast in soft PEG hydrogels to generate increasingly homogenous organoids of a highly defined shape that can be readily imaged and allow for downstream organoid selection (165). Using this system, the authors performed a cancer drug screen with 80 compounds using patient-derived colorectal cancer organoids and high content imaging. In this screen of 80 different drug compounds, three compounds exhibited clear phenotypic effects in high-content imaging that would not otherwise be found in a typical 2D drug screen. Future adaptation of organoid systems could leverage the ability of CRISPR-based tools to program patient-specific mutations or epigenetic states in conjunction with this drug screening approach to inform personalized medicine interventions.

## 5. OUTLOOK

### 5.1. Combinatorial Assembly of Genome Editing Machinery

The main limitation to the clinical translation of nonviral delivery methods has been their lower efficiency as compared to viral vectors. However, significant advances to address this limitation have been made through enhancements in biomaterial particle design, tissue targeting, and cellular uptake, combined with improved understanding of the delivery process. As formulations are developed that meet the thresholds for therapeutic efficacy, the advantages offered by biomaterial vectors may outweigh the increased viral efficiency in some cases (170). Such development will likely start in applications where viral vectors are particularly limiting, including applications with larger nucleic acid payloads. Other opportunities include settings where direct delivery of precomplexed RNP is advantageous, high-viral-dose requirements pose safety and production concerns, or therapy development and clinical trial costs are prohibitive. Hybrid strategies are also being developed where transgene-free virus-like particles deliver Cas9 protein directly (171, 172) and viruses are modified with biomaterials to improve their efficacy (173). Furthermore, Cas9 modification for use as a biologic, without any extra delivery vehicles, is showing promise, although still in development (36). As gene therapies expand to encompass more genetic diseases, we expect a diverse toolbox of delivery vehicles comprising viral, nonviral, and physical methods that will accommodate various delivery needs.

### 5.2. Informing CRISPR-Cas Targeting Strategies Using Biomaterials

While it is relatively easy to determine which gRNAs perform best in vitro, it is unclear if the same gRNAs will eventually be the most therapeutically effective in vivo. Gene editing activity with a specific gRNA is often first screened in easily transfected cell lines, yet the most efficient gRNA in this scenario might not translate to be the most effective when applied to other cell types in different cellular niches. Importantly, recent work in CRISPR-screening approaches with organoids has shown that only a fraction of gRNAs that were effective in transformed cell culture were also effective in more native-like organoid environments (163). Genomic editing outcomes in these more native-like contexts may be influenced through a variety of factors, including changes in chromatin structure, changes in mitotic state of the cells, and changes in active DNA repair pathways. Recently, the genome editing toolbox has expanded, with new strategies such as base editing and prime editing complementing genome and epigenome editing approaches (8, 9, 174, 175). The selection of these various CRISPR editing approaches may further be influenced by the biophysical microenvironment, where some strategies may prove to be more efficient than others when utilized in a more native-like microenvironment.

Over the past few years, many new insights have emerged for predicting the optimal gRNAs to use for CRISPR-Cas9 approaches based on potential off-target sites and other relevant genomic features of the on-target DNA site (176–183). From these systematic studies of Cas9 activity, we have learned that the chromatin context of the gRNA target site is an important modulator of gRNA activity. For example, numerous reports have indicated that Cas9 binding to its target DNA sequence can be restricted when the corresponding target sequence falls in the nucleosome dyad (184–186), and Cas9 off-target binding for the same gRNA can be markedly different between cell types (187). An additional consideration of gRNA targeting efficiency in vivo is that the biophysical microenvironment has been shown to be a strong modulator of chromatin accessibility and mobility (188–194). Therefore, culture on more native-like materials could promote chromatin mobility and protein turnover in a way that could increase the efficiency of various genome editing strategies, and this feature could be further leveraged to find gRNAs that would more likely be active in the tissue context.

The native microenvironment may also modulate the activity of DNA repair pathways that could result in drastically different performance of various CRISPR interventions depending on the culture scenario. HDR activity is generally low in nondividing cells, as it can only robustly occur in the S and G2 phases of the cell cycle (195). As the division rate of many cells can be strongly controlled by the biophysical cellular microenvironment (196), with division rates being the highest on rigid plastic substrates, it is likely that HDR may occur with reduced frequency in the softer in vivo environments. Additionally, the efficiency of DNA repair pathways is strongly altered by the microenvironment, such as when cells undergo migration through constricted pores (197), during hypoxia (198), and in other conditions. Recent work highlighted that HDR at DSBs results in nuclear actin polymerization and the mobilization of these breaks to the nuclear envelope to be repaired (199). Nuclear actin levels are tightly regulated by the extracellular environment (199, 200), so it is likely that these repair mechanisms can be modulated by the microenvironmental niche. While the mechanisms underlying many of these changes in DNA repair pathways are unknown, studying the influence of different biomaterial niches on the DNA repair process following gene editing will help answer these important questions and will likely result in new strategies to improve gene editing efficiency in vivo.

### 5.3. Biomaterial Strategies for Improving Epigenome Editing Outcomes

Epigenome editing outcomes with CRISPR-Cas9 may also be similarly influenced by the biomaterial microenvironment of the cell. Previous studies have highlighted how the success of various forms of somatic cell reprogramming can be increased by optimization of the biomaterial environment during the reprogramming process. In an elegant study, 3D culture in PEG hydrogels promoted pluripotency and improved iPSC reprogramming when compared to reprogramming on 2D hydrogels (201). High-throughput screening of various material formulations with varied stiffness, degradability, extracellular matrix protein presentation, and soluble factors in this 3D system identified a material formulation that was four times more efficient at generating Oct4+ reprogrammed colonies when compared to the same reprogramming on 2D culture. Similarly, several transdifferentiation protocols for converting fibroblasts directly to a differentiated cell type of interest leverage different biomaterial platforms to boost reprogramming efficiency (202–204). This reprogramming is likely increased in part by changes in chromatin mobility and protein localization in different material contexts, as mentioned above.

Epigenome editing approaches with CRISPR-Cas9 are also highly context specific (10); thus, epigenetic reprogramming in a more native-like environment may improve the functionality of various tools. This ability of biomaterials to influence reprogramming is also likely due to the fact that biomaterial inputs can be potent modulators of the epigenetic state of the cell (205–207). One mechanism that functions to control these epigenetic changes in response to the biophysical microenvironment is the process of mechanotransduction. During mechanotransduction, mechanical forces are readily converted into changes in the epigenetic state of the cell through a variety of mechanisms including mechanosensitive nuclear shuttling of transcriptional activators/repressors, changes in histone mark deposition, the activation of epigenetic remodeling complexes, and potentially direct deformations resulting in changes in transient chromatin accessibility (188, 200, 208–213).

Much of our knowledge on the function of the CRISPR-Cas9 system comes from studies of abnormal cell types cultured in abnormal conditions such as 2D monolayer culture on tissue culture plastic. Moving forward, new insights about genome editing will come from work in clinically relevant cell types using more complex in vitro model systems of the cellular microenvironment. Consequently, the use of new biomaterial technologies alongside advanced genome editing tools will dramatically improve the translation of biomedical research to advance human health.

## CRISPR GENOME EDITING TOOLS

Recent developments and expansion of CRISPR genome editing tools and variants have supported rapid advancement in several areas of biology, including target discovery, gene therapy, crop and animal modification, diagnostics, and molecular imaging (3–6). The main components of conventional CRISPR gene editing systems are a CRISPR-associated (Cas) nuclease and a guide RNA (gRNA), which complex together to form a ribonucleoprotein (RNP) complex. The gRNA contains a protospacer sequence that will direct the RNP complex to a specific nucleic acid target sequence, including genomic DNA. At the DNA target site, the RNP complex can then interact with the host genome and induce a double-stranded break (DSB) (7). Following this Cas-generated DSB, native cellular DNA repair programs take over and dictate the outcome of the genome editing process by initiating either nonhomologous end joining to join the ends of this break, or homology directed repair (HDR) using a repair template with homology to the target sequence. While nonhomologous end joining can be a useful repair pathway for genome editing, particularly gene knockout, many more opportunities are afforded by precise editing outcomes through HDR, which enables genome correction or insertion of additional sequences for gene modifications. Modified forms of Cas9, including the nuclease-deactivated dCas9, have further been important tools to target enzymes and other proteins to specific genomic regions and have led to different forms of gene editing, including base editing (8), prime editing (9), and epigenome editing for precise control of activation/repression (10, 11). Moreover, the development of other CRISPR-Cas systems such as Cas12, Cas13, and Cascade-Cas3 is enabling other forms of DNA and RNA editing (12).

## GENOME EDITING FOR THE DESIGN AND PRODUCTION OF NOVEL BIOMATERIALS

While this review largely focuses on biomaterials and genome editing for correcting and modeling human disease, there have been some recent developments wherein genome editing has been used to produce new biomaterial platforms. Excitingly, genome engineering allows for the modification of genes coding for natural biomaterials. These strategies contribute to a new branch of synthetic biology wherein these gene editing tools can be harnessed to create chimeric proteins or protein variants that can drastically change the material behavior and properties of the natural environment (13). Using these tools, engineered protein biopolymers can be designed and produced that have domains with different functionalities that modulate cell signaling, introduce novel amino acids or modifications, and provide inorganic binding domains for drug delivery (14). For example, smart biomaterial platforms have been created using purified Cas12a protein along with DNA-conjugated hydrogels, wherein Cas12a is used as a trigger for the release of various cargo, including cells and small molecules and proteins (15). Genome editing can also improve the biomanufacturing of biomaterials. Spider silk, which has a variety of desirable properties, can be mass produced in silk worms (16) or yeast (17) that have been genetically modified. Commercially, biomanufacturing offers a chance to generate new classes of materials inaccessible via conventional manufacturing methods. The facile production of libraries of thousands of variants of biomaterials from living organisms [or even in a cell-free manner (18)] enables rapid screening and identification of compositions with novel and improved functions. Recently, Zymergen has released Hyaline, a biopolymer film that was biofabricated in genome-edited living organisms (19). Hyaline has potential uses in touch-screen displays, circuit boards, and foldable electronics. As high-throughput genome editing technologies continue to advance alongside increased automation and machine learning, several novel biomanufactured materials will likely emerge in the coming years (20).

## References

[1] Wei T, Cheng Q, Farbiak L, Anderson DG, Langer R, Siegwart DJ. 2020. Delivery of tissue-targeted scalpels: opportunities and challenges for in vivo CRISPR/Cas-based genome editing. ACS Nano 14(8):9243–62 32697075 10.1021/acsnano.0c04707 PMC7996671

[2] Ringel T, Frey N, Ringnalda F, Janjuha S, Cherkaoui S, . 2020. Genome-scale CRISPR screening in human intestinal organoids identifies drivers of TGF-β resistance. Cell Stem Cell 26(3):431–40.e8 32142663 10.1016/j.stem.2020.02.007

[3] Fellmann C, Gowen BG, Lin P-C, Doudna JA, Corn JE. 2017. Cornerstones of CRISPR-Cas in drug discovery and therapy. Nat. Rev. Drug Discov 16(2):89–100 28008168 10.1038/nrd.2016.238 PMC5459481

[4] Gulei D, Raduly L, Berindan-Neagoe I, Calin GA. 2019. CRISPR-based RNA editing: diagnostic applications and therapeutic options. Expert Rev. Mol. Diagn 19(2):83–88 30632384 10.1080/14737159.2019.1568242

[5] Kumlehn J, Pietralla J, Hensel G, Pacher M, Puchta H. 2018. The CRISPR/Cas revolution continues: from efficient gene editing for crop breeding to plant synthetic biology. J. Integr. Plant Biol 60(12):1127–53 30387552 10.1111/jipb.12734

[6] Barrangou R, Doudna JA. 2016. Applications of CRISPR technologies in research and beyond. Nat. Biotechnol 34(9):933–41 27606440 10.1038/nbt.3659

[7] O’Connell MR, Oakes BL, Sternberg SH, East-Seletsky A, Kaplan M, Doudna JA. 2014. Programmable RNA recognition and cleavage by CRISPR/Cas9. Nature 516(7530):263–66 25274302 10.1038/nature13769 PMC4268322

[8] Gaudelli NM, Komor AC, Rees HA, Packer MS, Badran AH, 2017. Programmable base editing of A • T to G • C in genomic DNA without DNA cleavage. Nature 551(7681):464–71 29160308 10.1038/nature24644 PMC5726555

[9] Anzalone AV, Randolph PB, Davis JR, Sousa AA, Koblan LW, 2019. Search-and-replace genome editing without double-strand breaks or donor DNA. Nature 576(7785):149–57 31634902 10.1038/s41586-019-1711-4 PMC6907074

[10] Hilton IB, D’Ippolito AM, Vockley CM, Thakore PI, Crawford GE, 2015. Epigenome editing by a CRISPR-Cas9-based acetyltransferase activates genes from promoters and enhancers. Nat. Biotechnol 33(5):510–17 25849900 10.1038/nbt.3199 PMC4430400

[11] Thakore PI, Black JB, Hilton IB, Gersbach CA. 2016. Editing the epigenome: technologies for programmable transcription and epigenetic modulation. Nat. Methods 13(2):127–37 26820547 10.1038/nmeth.3733 PMC4922638

[12] Pickar-Oliver A, Gersbach CA. 2019. The next generation of CRISPR-Cas technologies and applications. Nat. Rev. Mol. Cell Biol 20(8):490–507 31147612 10.1038/s41580-019-0131-5 PMC7079207

[13] Gomes S, Leonor IB, Mano JF, Reis RL, Kaplan DL. 2012. Natural and genetically engineered proteins for tissue engineering. Prog. Polym. Sci 37(1):1–17 22058578 10.1016/j.progpolymsci.2011.07.003 PMC3207498

[14] Sengupta D, Heilshorn SC. 2010. Protein-engineered biomaterials: highly tunable tissue engineering scaffolds. Tissue Eng. Part B Rev 16(3):285–93 20141386 10.1089/ten.teb.2009.0591

[15] English MA, Soenksen LR, Gayet RV, de Puig H, Angenent-Mari NM, 2019. Programmable CRISPR-responsive smart materials. Science 365(6455):780–85 31439791 10.1126/science.aaw5122

[16] Xu J, Dong Q, Yu Y, Niu B, Ji D, 2018. Mass spider silk production through targeted gene replacement in *Bombyx mori*. PNAS 115(35):8757–62 30082397 10.1073/pnas.1806805115 PMC6126722

[17] Jansson R, Lau CH, Ishida T, Ramström M, Sandgren M, Hedhammar M. 2016. Functionalized silk assembled from a recombinant spider silk fusion protein (Z-4RepCT) produced in the methylotrophic yeast *Pichia pastoris*. Biotechnol. J 11(5):687–99 26814048 10.1002/biot.201500412

[18] Kelwick RJR, Webb AJ, Freemont PS. 2020. Biological materials: the next frontier for cell-free synthetic biology. Front. Bioeng. Biotechnol 8:399 32478045 10.3389/fbioe.2020.00399 PMC7235315

[19] Cumbers J. 2020. Inspired by nature, Zymergen brews high-performance bio-electronics. Forbes, Apr. 12. https://www.forbes.com/sites/johncumbers/2020/04/12/inspired-by-nature-zymergen-brews-high-performance-bio-electronics/#2077a9ab2f18

[20] Le Feuvre RA, Scrutton NS. 2018. A living foundry for synthetic biological materials: a synthetic biology roadmap to new advanced materials. Synth. Syst. Biotechnol 3(2):105–12 29900423 10.1016/j.synbio.2018.04.002 PMC5995479

[21] Dalkara D, Byrne LC, Klimczak RR, Visel M, Yin L, 2013. In vivo-directed evolution of a new adeno-associated virus for therapeutic outer retinal gene delivery from the vitreous. Sci. Transl. Med 5(189):189ra76 10.1126/scitranslmed.3005708 23761039

[22] van Haasteren J, Li J, Scheideler OJ, Murthy N, Schaffer DV. 2020. The delivery challenge: fulfilling the promise of therapeutic genome editing. Nat. Biotechnol 38(7):845–55 32601435 10.1038/s41587-020-0565-5

[23] Mingozzi F, High KA. 2017. Overcoming the host immune response to adeno-associated virus gene delivery vectors: the race between clearance, tolerance, neutralization, and escape. Annu. Rev. Virol 4:511–34 28961410 10.1146/annurev-virology-101416-041936

[24] Charlesworth CT, Deshpande PS, Dever DP, Camarena J, Lemgart VT, 2019. Identification of preexisting adaptive immunity to Cas9 proteins in humans. Nat. Med 25(2):249–54 30692695 10.1038/s41591-018-0326-x PMC7199589

[25] Nelson CE, Wu Y, Gemberling MP, Oliver ML, Waller MA, . 2019. Long-term evaluation of AAV-CRISPR genome editing for Duchenne muscular dystrophy. Nat. Med 25(3):427–32 30778238 10.1038/s41591-019-0344-3 PMC6455975

[26] Li L, Hu S, Chen X. 2018. Non-viral delivery systems for CRISPR/Cas9-based genome editing: challenges and opportunities. Biomaterials 171:207–18 29704747 10.1016/j.biomaterials.2018.04.031 PMC5944364

[27] Goswami R, Subramanian G, Silayeva L, Newkirk I, Doctor D, 2019. Gene therapy leaves a vicious cycle. Front. Oncol 9:297 31069169 10.3389/fonc.2019.00297 PMC6491712

[28] Rui Y, Wilson DR, Green JJ. 2019. Non-viral delivery to enable genome editing. Trends Biotechnol. 37(3):281–93 30278987 10.1016/j.tibtech.2018.08.010 PMC6378131

[29] Nelson CE, Gersbach CA. 2016. Engineering delivery vehicles for genome editing. Annu. Rev. Chem. Biomol. Eng 7:637–62 27146557 10.1146/annurev-chembioeng-080615-034711

[30] Stewart MP, Lorenz A, Dahlman J, Sahay G. 2016. Challenges in carrier-mediated intracellular delivery: moving beyond endosomal barriers. WIREs Nanomed. Nanobiotechnol 8(3):465–78 10.1002/wnan.1377 26542891

[31] Lino CA, Harper JC, Carney JP, Timlin JA. 2018. Delivering CRISPR: a review of the challenges and approaches. Drug Deliv. 25(1):1234–57 29801422 10.1080/10717544.2018.1474964 PMC6058482

[32] Tong S, Moyo B, Lee CM, Leong K, Bao G. 2019. Engineered materials for in vivo delivery of genome-editing machinery. Nat. Rev. Mater 4(11):726–37 34094589 10.1038/s41578-019-0145-9 PMC8174554

[33] Chew WL, Tabebordbar M, Cheng JKW, Mali P, Wu EY, 2016. A multi-functional AAV-CRISPR-Cas9 and its host response. Nat. Methods 13(10):868–74 27595405 10.1038/nmeth.3993 PMC5374744

[34] Carlson-Stevermer J, Abdeen AA, Kohlenberg L, Goedland M, Molugu K, 2017. Assembly of CRISPR ribonucleoproteins with biotinylated oligonucleotides via an RNA aptamer for precise gene editing. Nat. Commun 8(1):1711 29167458 10.1038/s41467-017-01875-9 PMC5700129

[35] Hendel A, Bak RO, Clark JT, Kennedy AB, Ryan DE, 2015. Chemically modified guide RNAs enhance CRISPR-Cas genome editing in human primary cells. Nat. Biotechnol 33(9):985–89 26121415 10.1038/nbt.3290 PMC4729442

[36] Lim D, Sreekanth V, Cox KJ, Law BK, Wagner BK, 2020. Engineering designer beta cells with a CRISPR-Cas9 conjugation platform. Nat. Commun 11(1):4043 32792475 10.1038/s41467-020-17725-0 PMC7426819

[37] Vakulskas CA, Behlke MA. 2019. Evaluation and reduction of CRISPR off-target cleavage events. Nucleic Acid Ther. 29(4):167–74 31107154 10.1089/nat.2019.0790 PMC6686686

[38] Toy R, Hayden E, Shoup C, Baskaran H, Karathanasis E. 2011. Effect of particle size, density and shape on margination of nanoparticles in microcirculation. Nanotechnology 22(11):115101 21387846 10.1088/0957-4484/22/11/115101 PMC3530262

[39] Alexis F, Pridgen E, Molnar LK, Farokhzad OC. 2008. Factors affecting the clearance and biodistribution of polymeric nanoparticles. Mol. Pharm 5(4):505–15 18672949 10.1021/mp800051m PMC2663893

[40] Hoshyar N, Gray S, Han H, Bao G. 2016. The effect of nanoparticle size on in vivo pharmacokinetics and cellular interaction. Nanomedicine 11(6):673–92 27003448 10.2217/nnm.16.5 PMC5561790

[41] Duan X, Li Y. 2013. Physicochemical characteristics of nanoparticles affect circulation, biodistribution, cellular internalization, and trafficking. Small 9(9–10):1521–32 23019091 10.1002/smll.201201390

[42] Jiang W, Kim BYS, Rutka JT, Chan WCW. 2008. Nanoparticle-mediated cellular response is size-dependent. Nat. Nanotechnol 3(3):145–50 18654486 10.1038/nnano.2008.30

[43] Kinnear C, Moore TL, Rodriguez-Lorenzo L, Rothen-Rutishauser B, Petri-Fink A. 2017. Form follows function: nanoparticle shape and its implications for nanomedicine. Chem. Rev 117(17):11476–521 28862437 10.1021/acs.chemrev.7b00194

[44] Jo DH, Kim JH, Lee TG, Kim JH. 2015. Size, surface charge, and shape determine therapeutic effects of nanoparticles on brain and retinal diseases. Nanomed. Nanotechnol. Biol. Med 11(7):1603–11 10.1016/j.nano.2015.04.015 25989200

[45] Wang P, Zhang L, Zheng W, Cong L, Guo Z, 2018. Thermo-triggered release of CRISPR-Cas9 system by lipid-encapsulated gold nanoparticles for tumor therapy. Angew. Chem. Int. Ed 57(6):1491–96 10.1002/anie.201708689 29282854

[46] Caldorera-Moore M, Guimard N, Shi L, Roy K. 2010. Designer nanoparticles: incorporating size, shape, and triggered release into nanoscale drug carriers. Expert Opin. Drug Deliv 7(4):479–95 20331355 10.1517/17425240903579971 PMC2845970

[47] Cai W, Luo T, Mao L, Wang M. 2020. Spatiotemporal delivery of CRISPR/Cas9 genome editing machinery using stimuli-responsive vehicles. Angew. Chem. Int. Ed 60(16):8596–606 10.1002/anie.202005644 32385892

[48] Wu Y, Zheng J, Zeng Q, Zhang T, Xing D. 2020. Light-responsive charge-reversal nanovector for high-efficiency in vivo CRISPR/Cas9 gene editing with controllable location and time. Nano Res. 13(9):2399–406

[49] Yoo J, Park C, Yi G, Lee D, Koo H. 2019. Active targeting strategies using biological ligands for nanoparticle drug delivery systems. Cancers 11(5):640 31072061 10.3390/cancers11050640 PMC6562917

[50] Zhen S, Li X. 2020. Liposomal delivery of CRISPR/Cas9. Cancer Gene Ther. 27(7):515–27 31676843 10.1038/s41417-019-0141-7

[51] Guidotti G, Brambilla L, Rossi D. 2017. Cell-penetrating peptides: from basic research to clinics. Trends Pharmacol. Sci 38(4):406–24 28209404 10.1016/j.tips.2017.01.003

[52] Shi N-Q, Qi X-R, Xiang B, Zhang Y. 2014. A survey on “Trojan Horse” peptides: opportunities, issues and controlled entry to “Troy.” J. Control. Release 194:53–70 25151981 10.1016/j.jconrel.2014.08.014

[53] Park H, Oh J, Shim G, Cho B, Chang Y, 2019. In vivo neuronal gene editing via CRISPR-Cas9 amphiphilic nanocomplexes alleviates deficits in mouse models of Alzheimer’s disease. Nat. Neurosci 22(4):524–28 30858603 10.1038/s41593-019-0352-0

[54] Ramakrishna S, Kwaku Dad A-B, Beloor J, Gopalappa R, Lee S-K, Kim H. 2014. Gene disruption by cell-penetrating peptide-mediated delivery of Cas9 protein and guide RNA. Genome Res. 24(6):1020–27 24696462 10.1101/gr.171264.113 PMC4032848

[55] Lobba MJ, Fellmann C, Marmelstein AM, Maza JC, Kissman EN, 2020. Site-specific bioconjugation through enzyme-catalyzed tyrosine-cysteine bond formation. ACS Cent. Sci 6(9):1564–71 32999931 10.1021/acscentsci.0c00940 PMC7517114

[56] Narayanan K, Yen SK, Dou Q, Padmanabhan P, Sudhaharan T, 2013. Mimicking cellular transport mechanism in stem cells through endosomal escape of new peptide-coated quantum dots. Sci. Rep 3:2184 23851637 10.1038/srep02184 PMC3711047

[57] Chen G, Abdeen AA, Wang Y, Shahi PK, Robertson S, 2019. A biodegradable nanocapsule delivers a Cas9 ribonucleoprotein complex for in vivo genome editing. Nat. Nanotechnol 14(10):974–80 31501532 10.1038/s41565-019-0539-2 PMC6778035

[58] Green JJ, Langer R, Anderson DG. 2008. A combinatorial polymer library approach yields insight into nonviral gene delivery. Acc. Chem. Res 41(6):749–59 18507402 10.1021/ar7002336 PMC3490629

[59] Dahlman JE, Kauffman KJ, Xing Y, Shaw TE, Mir FF, 2017. Barcoded nanoparticles for high throughput in vivo discovery of targeted therapeutics. PNAS 114(8):2060–65 28167778 10.1073/pnas.1620874114 PMC5338412

[60] Sago CD, Lokugamage MP, Paunovska K, Vanover DA, Monaco CM, 2018. High-throughput in vivo screen of functional mRNA delivery identifies nanoparticles for endothelial cell gene editing. PNAS 115(42):E9944–52 30275336 10.1073/pnas.1811276115 PMC6196543

[61] Chew WL. 2018. Immunity to CRISPR Cas9 and Cas12a therapeutics. WIREs Syst. Biol. Med 10(1):e1408 10.1002/wsbm.1408 29083112

[62] Crudele JM, Chamberlain JS. 2018. Cas9 immunity creates challenges for CRISPR gene editing therapies. Nat. Commun 9(1):3497 30158648 10.1038/s41467-018-05843-9 PMC6115392

[63] Li A, Tanner MR, Lee CM, Hurley AE, Giorgi MD, 2020. AAV-CRISPR gene editing is negated by pre-existing immunity to Cas9. Mol. Ther 28(6):1432–41 32348718 10.1016/j.ymthe.2020.04.017 PMC7264438

[64] Fadeel B. 2019. Hide and seek: nanomaterial interactions with the immune system. Front. Immunol 10:133 30774634 10.3389/fimmu.2019.00133 PMC6367956

[65] O’Brien K, Breyne K, Ughetto S, Laurent LC, Breakefield XO. 2020. RNA delivery by extracellular vesicles in mammalian cells and its applications. Nat. Rev. Mol. Cell Biol 21:585–606 32457507 10.1038/s41580-020-0251-y PMC7249041

[66] Rodriguez PL, Harada T, Christian DA, Pantano DA, Tsai RK, Discher DE. 2013. Minimal “self” peptides that inhibit phagocytic clearance and enhance delivery of nanoparticles. Science 339(6122):971–75 23430657 10.1126/science.1229568 PMC3966479

[67] Lee K, Conboy M, Park HM, Jiang F, Kim HJ, 2017. Nanoparticle delivery of Cas9 ribonucleoprotein and donor DNA in vivo induces homology-directed DNA repair. Nat. Biomed. Eng 1(11):889–901 29805845 10.1038/s41551-017-0137-2 PMC5968829

[68] Hsieh Y-C, Wang H-E, Lin W-W, Roffler SR, Cheng T-C, 2018. Pre-existing anti-polyethylene glycol antibody reduces the therapeutic efficacy and pharmacokinetics of PEGylated liposomes. Theranostics 8(11):3164–75 29896310 10.7150/thno.22164 PMC5996368

[69] Verhoef JJF, Carpenter JF, Anchordoquy TJ, Schellekens H. 2014. Potential induction of anti-PEG antibodies and complement activation toward PEGylated therapeutics. Drug Discov. Today 19(12):1945–52 25205349 10.1016/j.drudis.2014.08.015

[70] Van Haute D, Berlin JM. 2017. Challenges in realizing selectivity for nanoparticle biodistribution and clearance: lessons from gold nanoparticles. Ther. Deliv 8(9):763–74 28825391 10.4155/tde-2017-0057 PMC6123877

[71] Schluck M, Hammink R, Figdor CG, Verdoes M, Weiden J. 2019. Biomaterial-based activation and expansion of tumor-specific T cells. Front. Immunol 10:931 31130945 10.3389/fimmu.2019.00931 PMC6509561

[72] Monette A, Ceccaldi C, Assaad E, Lerouge S, Lapointe R. 2016. Chitosan thermogels for local expansion and delivery of tumor-specific T lymphocytes towards enhanced cancer immunotherapies. Biomaterials 75:237–49 26513416 10.1016/j.biomaterials.2015.10.021

[73] Heathman TRJ, Nienow AW, McCall MJ, Coopman K, Kara B, Hewitt CJ. 2015. The translation of cell-based therapies: clinical landscape and manufacturing challenges. Regen. Med 10(1):49–64 25562352 10.2217/rme.14.73

[74] Zakrzewski W, Dobrzyński M, Szymonowicz M, Rybak Z. 2019. Stem cells: past, present, and future. Stem Cell Res. Ther 10:68 30808416 10.1186/s13287-019-1165-5 PMC6390367

[75] Salg GA, Giese NA, Schenk M, Hüttner FJ, Felix K, 2019. The emerging field of pancreatic tissue engineering: a systematic review and evidence map of scaffold materials and scaffolding techniques for insulin-secreting cells. J. Tissue Eng 10.1177/2041731419884708 PMC6823987 31700597

[76] Rohaan MW, Wilgenhof S, Haanen JBAG. 2019. Adoptive cellular therapies: the current landscape. Virchows Arch. 474(4):449–61 30470934 10.1007/s00428-018-2484-0 PMC6447513

[77] Facklam AL, Volpatti LR, Anderson DG. 2020. Biomaterials for personalized cell therapy. Adv. Mater 32(13):1902005 10.1002/adma.201902005 31495970

[78] Palucka AK, Coussens LM. 2016. The basis of oncoimmunology. Cell 164(6):1233–47 26967289 10.1016/j.cell.2016.01.049 PMC4788788

[79] Ribas A. 2015. Releasing the brakes on cancer immunotherapy. N. Engl. J. Med 373:1490–92 26348216 10.1056/NEJMp1510079

[80] Holzinger A, Abken H. 2020. Advances and challenges of CAR T cells in clinical trials. Recent Results Cancer Res. 214:93–128 31473850 10.1007/978-3-030-23765-3_3

[81] Piscopo NJ, Mueller KP, Das A, Hematti P, Murphy WL, 2018. Bioengineering solutions for manufacturing challenges in CAR T cells. Biotechnol. J 13(2):1700095 10.1002/biot.201700095 PMC5796845 28840981

[82] Jin C, Fotaki G, Ramachandran M, Nilsson B, Essand M, Yu D. 2016. Safe engineering of CAR T cells for adoptive cell therapy of cancer using long-term episomal gene transfer. EMBO Mol. Med 8(7):702–11 27189167 10.15252/emmm.201505869 PMC4931286

[83] Roth TL, Puig-Saus C, Yu R, Shifrut E, Carnevale J, 2018. Reprogramming human T cell function and specificity with non-viral genome targeting. Nature 559(7714):405–9 29995861 10.1038/s41586-018-0326-5 PMC6239417

[84] Nguyen DN, Roth TL, Li PJ, Chen PA, Apathy R, 2020. Polymer-stabilized Cas9 nanoparticles and modified repair templates increase genome editing efficiency. Nat. Biotechnol 38(1):44–49 31819258 10.1038/s41587-019-0325-6 PMC6954310

[85] Olden BR, Cheng Y, Yu JL, Pun SH. 2018. Cationic polymers for non-viral gene delivery to human T cells. J. Control. Release 282:140–47 29518467 10.1016/j.jconrel.2018.02.043 PMC6008197

[86] O’Connor RS, Hao X, Shen K, Bashour K, Akimova T, 2012. Substrate rigidity regulates human T cell activation and proliferation. J. Immunol 189(3):1330–39 22732590 10.4049/jimmunol.1102757 PMC3401283

[87] Hammink R, Mandal S, Eggermont LJ, Nooteboom M, Willems PHGM, 2017. Controlling T-cell activation with synthetic dendritic cells using the multivalency effect. ACS Omega 2(3):937–45 28393131 10.1021/acsomega.6b00436 PMC5377267

[88] Cheung AS, Zhang DKY, Koshy ST, Mooney DJ. 2018. Scaffolds that mimic antigen-presenting cells enable ex vivo expansion of primary T cells. Nat. Biotechnol 36(2):160–69 29334370 10.1038/nbt.4047 PMC5801009

[89] Abdeen AA, Saha K. 2017. Manufacturing cell therapies using engineered biomaterials. Trends Biotechnol. 35(10):971–82 28711155 10.1016/j.tibtech.2017.06.008 PMC5621598

[90] Stephan MT, Moon JJ, Um SH, Bershteyn A, Irvine DJ. 2010. Therapeutic cell engineering with surface-conjugated synthetic nanoparticles. Nat. Med 16(9):1035–41 20711198 10.1038/nm.2198 PMC2935928

[91] Huang B, Abraham WD, Zheng Y, Bustamante López SC, Luo SS, Irvine DJ. 2015. Active targeting of chemotherapy to disseminated tumors using nanoparticle-carrying T cells. Sci. Transl. Med 7(291):291ra94 10.1126/scitranslmed.aaa5447 PMC4687972 26062846

[92] Smith TT, Moffett HF, Stephan SB, Opel CF, Dumigan AG, 2017. Biopolymers codelivering engineered T cells and STING agonists can eliminate heterogeneous tumors. J. Clin. Investig 127(6):2176–91 28436934 10.1172/JCI87624 PMC5451231

[93] Stephan SB, Taber AM, Jileaeva I, Pegues EP, Sentman CL, Stephan MT. 2015. Biopolymer implants enhance the efficacy of adoptive T-cell therapy. Nat. Biotechnol 33(1):97–101 25503382 10.1038/nbt.3104 PMC4289408

[94] Coon ME, Stephan SB, Gupta V, Kealey CP, Stephan MT. 2020. Nitinol thin films functionalized with CAR-T cells for the treatment of solid tumours. Nat. Biomed. Eng 4(2):195–206 31819155 10.1038/s41551-019-0486-0

[95] Smith TT, Stephan SB, Moffett HF, McKnight LE, Ji W, 2017. In situ programming of leukaemia-specific T cells using synthetic DNA nanocarriers. Nat. Nanotechnol 12(8):813–20 28416815 10.1038/nnano.2017.57 PMC5646367

[96] Borowicz S, Van Scoyk M, Avasarala S, Karuppusamy Rathinam MK, Tauler J, 2014. The soft agar colony formation assay. J. Vis. Exp 92:e51998 10.3791/51998 PMC4353381 25408172

[97] Petersen OW, Ronnov-Jessen L, Howlett AR, Bissell MJ. 1992. Interaction with basement membrane serves to rapidly distinguish growth and differentiation pattern of normal and malignant human breast epithelial cells. PNAS 89(19):9064–68 1384042 10.1073/pnas.89.19.9064 PMC50065

[98] Barcellos-Hoff MH, Aggeler J, Ram TG, Bissell MJ. 1989. Functional differentiation and alveolar morphogenesis of primary mammary cultures on reconstituted basement membrane. Development 105(2):223–35 2806122 10.1242/dev.105.2.223 PMC2948482

[99] Badeau BA, Comerford MP, Arakawa CK, Shadish JA, DeForest CA. 2018. Engineered modular biomaterial logic gates for environmentally triggered therapeutic delivery. Nat. Chem 10(3):251–58 29461528 10.1038/nchem.2917 PMC5822735

[100] Khetan S, Guvendiren M, Legant WR, Cohen DM, Chen CS, Burdick JA. 2013. Degradation-mediated cellular traction directs stem cell fate in covalently crosslinked three-dimensional hydrogels. Nat. Mater 12(5):458–65 23524375 10.1038/nmat3586 PMC3633615

[101] Baker BM, Trappmann B, Wang WY, Sakar MS, Kim IL, 2015. Cell-mediated fibre recruitment drives extracellular matrix mechanosensing in engineered fibrillar microenvironments. Nat. Mater 14(12):1262–68 26461445 10.1038/nmat4444 PMC4654682

[102] Madl CM, LeSavage BL, Dewi RE, Dinh CB, Stowers RS, 2017. Maintenance of neural progenitor cell stemness in 3D hydrogels requires matrix remodelling. Nat. Mater 16(12):1233–42 29115291 10.1038/nmat5020 PMC5708569

[103] Tibbitt MW, Anseth KS. 2009. Hydrogels as extracellular matrix mimics for 3D cell culture. Biotechnol. Bioeng 103(4):655–63 19472329 10.1002/bit.22361 PMC2997742

[104] Simian M, Bissell MJ. 2017. Organoids: a historical perspective of thinking in three dimensions. J. Cell Biol 216(1):31–40 28031422 10.1083/jcb.201610056 PMC5223613

[105] Sato T, Vries RG, Snippert HJ, van de Wetering M, Barker N, 2009. Single Lgr5 stem cells build crypt-villus structures in vitro without a mesenchymal niche. Nature 459(7244):262–65 19329995 10.1038/nature07935

[106] Eiraku M, Takata N, Ishibashi H, Kawada M, Sakakura E, 2011. Self-organizing optic-cup morphogenesis in three-dimensional culture. Nature 472(7341):51–56 21475194 10.1038/nature09941

[107] Eiraku M, Watanabe K, Matsuo-Takasaki M, Kawada M, Yonemura S, 2008. Self-organized formation of polarized cortical tissues from ESCs and its active manipulation by extrinsic signals. Cell Stem Cell 3(5):519–32 18983967 10.1016/j.stem.2008.09.002

[108] Muguruma K, Nishiyama A, Kawakami H, Hashimoto K, Sasai Y. 2015. Self-organization of polarized cerebellar tissue in 3D culture of human pluripotent stem cells. Cell Rep. 10(4):537–50 25640179 10.1016/j.celrep.2014.12.051

[109] Lancaster MA, Renner M, Martin C-A, Wenzel D, Bicknell LS, 2013. Cerebral organoids model human brain development and microcephaly. Nature 501(7467):373–79 23995685 10.1038/nature12517 PMC3817409

[110] Takebe T, Sekine K, Enomura M, Koike H, Kimura M, 2013. Vascularized and functional human liver from an iPSC-derived organ bud transplant. Nature 499(7459):481–84 23823721 10.1038/nature12271

[111] Matano M, Date S, Shimokawa M, Takano A, Fujii M, 2015. Modeling colorectal cancer using CRISPR-Cas9-mediated engineering of human intestinal organoids. Nat. Med 21(3):256–62 25706875 10.1038/nm.3802

[112] Rossi G, Manfrin A, Lutolf MP. 2018. Progress and potential in organoid research. Nat. Rev. Genet 19(11):671–87 30228295 10.1038/s41576-018-0051-9

[113] Caliari SR, Burdick JA. 2016. A practical guide to hydrogels for cell culture. Nat. Methods 13(5):405–14 27123816 10.1038/nmeth.3839 PMC5800304

[114] Hughes CS, Postovit LM, Lajoie GA. 2010. Matrigel: a complex protein mixture required for optimal growth of cell culture. Proteomics 10(9):1886–90 20162561 10.1002/pmic.200900758

[115] Aisenbrey EA, Murphy WL. 2020. Synthetic alternatives to Matrigel. Nat. Rev. Mater 5(7):539–51 32953138 10.1038/s41578-020-0199-8 PMC7500703

[116] Han WM, Anderson SE, Mohiuddin M, Barros D, Nakhai SA, 2018. Synthetic matrix enhances transplanted satellite cell engraftment in dystrophic and aged skeletal muscle with comorbid trauma. Sci. Adv 4(8):eaar4008 10.1126/sciadv.aar4008 PMC6093653 30116776

[117] Gjorevski N, Sachs N, Manfrin A, Giger S, Bragina ME, 2016. Designer matrices for intestinal stem cell and organoid culture. Nature 539(7630):560–64 27851739 10.1038/nature20168

[118] Hui E, Gimeno KI, Guan G, Caliari SR. 2019. Spatiotemporal control of viscoelasticity in phototunable hyaluronic acid hydrogels. Biomacromolecules 20(11):4126–34 31600072 10.1021/acs.biomac.9b00965 PMC7329014

[119] Nam S, Stowers R, Lou J, Xia Y, Chaudhuri O. 2019. Varying PEG density to control stress relaxation in alginate-PEG hydrogels for 3D cell culture studies. Biomaterials 200:15–24 30743050 10.1016/j.biomaterials.2019.02.004 PMC6463514

[120] Chaudhuri O, Gu L, Klumpers D, Darnell M, Bencherif SA, 2016. Hydrogels with tunable stress relaxation regulate stem cell fate and activity. Nat. Mater 15(3):326–34 26618884 10.1038/nmat4489 PMC4767627

[121] Chaudhuri O. 2017. Viscoelastic hydrogels for 3D cell culture. Biomater. Sci 5(8):1480–90 28584885 10.1039/c7bm00261k

[122] Lee JY, Chang JK, Dominguez AA, Lee H-P, Nam S, 2019. YAP-independent mechanotransduction drives breast cancer progression. Nat. Commun 10(1):1848 31015465 10.1038/s41467-019-09755-0 PMC6478686

[123] Lou J, Stowers R, Nam S, Xia Y, Chaudhuri O. 2018. Stress relaxing hyaluronic acid-collagen hydrogels promote cell spreading, fiber remodeling, and focal adhesion formation in 3D cell culture. Biomaterials 154:213–22 29132046 10.1016/j.biomaterials.2017.11.004

[124] Veltman JA, Brunner HG. 2012. De novo mutations in human genetic disease. Nat. Rev. Genet 13(8):565–75 22805709 10.1038/nrg3241

[125] Bauer-Mehren A, Rautschka M, Sanz F, Furlong LI. 2010. DisGeNET: a Cytoscape plugin to visualize, integrate, search and analyze gene-disease networks. Bioinformatics 26(22):2924–26 20861032 10.1093/bioinformatics/btq538

[126] Cornish AJ, Filippis I, David A, Sternberg MJE. 2015. Exploring the cellular basis of human disease through a large-scale mapping of deleterious genes to cell types. Genome Med. 7:95 26330083 10.1186/s13073-015-0212-9 PMC4557825

[127] Gopal S, Rodrigues AL, Dordick JS. 2020. Exploiting CRISPR Cas9 in three-dimensional stem cell cultures to model disease. Front. Bioeng. Biotechnol 8:692 32671050 10.3389/fbioe.2020.00692 PMC7326781

[128] Fusco P, Parisatto B, Rampazzo E, Persano L, Frasson C, 2019. Patient-derived organoids (PDOs) as a novel in vitro model for neuroblastoma tumours. BMC Cancer 19(1):970 31638925 10.1186/s12885-019-6149-4 PMC6802324

[129] Nagle PW, Plukker JTM, Muijs CT, van Luijk P, Coppes RP. 2018. Patient-derived tumor organoids for prediction of cancer treatment response. Semin. Cancer Biol 53:258–64 29966678 10.1016/j.semcancer.2018.06.005

[130] Campbell PJ, Getz G, Korbel JO, Stuart JM, Jennings JL, (ICGC/TCGA Pan-Cancer Anal. Whole Genomes Consort.). 2020. Pan-cancer analysis of whole genomes. Nature 578:82–9332025007

[131] Greenman C, Stephens P, Smith R, Dalgliesh GL, Hunter C, 2007. Patterns of somatic mutation in human cancer genomes. Nature 446(7132):153–58 17344846 10.1038/nature05610 PMC2712719

[132] Bladen CL, Salgado D, Monges S, Foncuberta ME, Kekou K, 2015. The TREAT-NMD DMD Global Database: analysis of more than 7,000 Duchenne muscular dystrophy mutations. Hum. Mutat 36(4):395–402 25604253 10.1002/humu.22758 PMC4405042

[133] Nelson CE, Gersbach CA. 2019. Genome editing for Duchenne muscular dystrophy. In Muscle Gene Therapy, ed. Duan D, Mendell J, pp. 383–403. Cham, Switz.: Springer

[134] Schwank G, Koo B-K, Sasselli V, Dekkers JF, Heo I, 2013. Functional repair of CFTR by CRISPR/Cas9 in intestinal stem cell organoids of cystic fibrosis patients. Cell Stem Cell 13(6):653–58 24315439 10.1016/j.stem.2013.11.002

[135] Drost J, van Boxtel R, Blokzijl F, Mizutani T, Sasaki N, 2017. Use of CRISPR-modified human stem cell organoids to study the origin of mutational signatures in cancer. Science 358(6360):234–38 28912133 10.1126/science.aao3130 PMC6038908

[136] Matano M, Date S, Shimokawa M, Takano A, Fujii M, 2015. Modeling colorectal cancer using CRISPR-Cas9–mediated engineering of human intestinal organoids. Nat. Med 21:256–62 25706875 10.1038/nm.3802

[137] Mariani J, Coppola G, Zhang P, Abyzov A, Provini L, 2015. FOXG1-dependent dysregulation of GABA/glutamate neuron differentiation in autism spectrum disorders. Cell 162(2):375–90 26186191 10.1016/j.cell.2015.06.034 PMC4519016

[138] van de Wetering M, Francies HE, Francis JM, Bounova G, Iorio F, 2015. Prospective derivation of a living organoid biobank of colorectal cancer patients. Cell 161(4):933–45 25957691 10.1016/j.cell.2015.03.053 PMC6428276

[139] Driehuis E, Clevers H. 2017. CRISPR/Cas 9 genome editing and its applications in organoids. Am. J. Physiol. Gastrointest. Liver Physiol 312(3):G257–65 28126704 10.1152/ajpgi.00410.2016

[140] Fujii M, Clevers H, Sato T. 2019. Modeling human digestive diseases with CRISPR-Cas9-modified organoids. Gastroenterology 156(3):562–76 30476497 10.1053/j.gastro.2018.11.048

[141] Wang T, Wei JJ, Sabatini DM, Lander ES. 2014. Genetic screens in human cells using the CRISPR-Cas9 system. Science 343(6166):80–84 24336569 10.1126/science.1246981 PMC3972032

[142] Shalem O, Sanjana NE, Hartenian E, Shi X, Scott DA, 2014. Genome-scale CRISPR-Cas9 knockout screening in human cells. Science 343(6166):84–87 24336571 10.1126/science.1247005 PMC4089965

[143] Gilbert LA, Horlbeck MA, Adamson B, Villalta JE, Chen Y, 2014. Genome-scale CRISPR-mediated control of gene repression and activation. Cell 159(3):647–61 25307932 10.1016/j.cell.2014.09.029 PMC4253859

[144] Klann TS, Black JB, Chellappan M, Safi A, Song L, 2017. CRISPR-Cas9 epigenome editing enables high-throughput screening for functional regulatory elements in the human genome. Nat. Biotechnol 35(6):561–68 28369033 10.1038/nbt.3853 PMC5462860

[145] Klann TS, Black JB, Gersbach CA. 2018. CRISPR-based methods for high-throughput annotation of regulatory DNA. Curr. Opin. Biotechnol 52:32–41 29500989 10.1016/j.copbio.2018.02.004 PMC6082715

[146] Xie S, Duan J, Li B, Zhou P, Hon GC. 2017. Multiplexed engineering and analysis of combinatorial enhancer activity in single cells. Mol. Cell 66(2):285–99.e5 28416141 10.1016/j.molcel.2017.03.007

[147] Dixit A, Parnas O, Li B, Chen J, Fulco CP, 2016. Perturb-seq: dissecting molecular circuits with scalable single-cell RNA profiling of pooled genetic screens. Cell 167(7):1853–66.e17 27984732 10.1016/j.cell.2016.11.038 PMC5181115

[148] Parnas O, Jovanovic M, Eisenhaure TM, Herbst RH, Dixit A, 2015. A genome-wide CRISPR screen in primary immune cells to dissect regulatory networks. Cell 162(3):675–86 26189680 10.1016/j.cell.2015.06.059 PMC4522370

[149] Doench JG. 2018. Am I ready for CRISPR? A user’s guide to genetic screens. Nat. Rev. Genet 19(2):67–80 29199283 10.1038/nrg.2017.97

[150] Sanjana NE, Wright J, Zheng K, Shalem O, Fontanillas P, 2016. High-resolution interrogation of functional elements in the noncoding genome. Science 353(6307):1545–49 27708104 10.1126/science.aaf7613 PMC5144102

[151] Fulco CP, Munschauer M, Anyoha R, Munson G, Grossman SR, 2016. Systematic mapping of functional enhancer-promoter connections with CRISPR interference. Science 354(6313):769–73 27708057 10.1126/science.aag2445 PMC5438575

[152] Chen S, Sanjana NE, Zheng K, Shalem O, Lee K, 2015. Genome-wide CRISPR screen in a mouse model of tumor growth and metastasis. Cell 160(6):1246–60 25748654 10.1016/j.cell.2015.02.038 PMC4380877

[153] Patel SJ, Sanjana NE, Kishton RJ, Eidizadeh A, Vodnala SK, 2017. Identification of essential genes for cancer immunotherapy. Nature 548(7669):537–42 28783722 10.1038/nature23477 PMC5870757

[154] Manguso RT, Pope HW, Zimmer MD, Brown FD, Yates KB, 2017. In vivo CRISPR screening identifies *Ptpn2* as a cancer immunotherapy target. Nature 547(7664):413–18 28723893 10.1038/nature23270 PMC5924693

[155] Braun CJ, Bruno PM, Horlbeck MA, Gilbert LA, Weissman JS, Hemann MT. 2016. Versatile in vivo regulation of tumor phenotypes by dCas9-mediated transcriptional perturbation. PNAS 113(27):E3892–900 27325776 10.1073/pnas.1600582113 PMC4941480

[156] Katigbak A, Cencic R, Robert F, Sénécha P, Scuoppo C, Pelletier J. 2016. A CRISPR/Cas9 functional screen identifies rare tumor suppressors. Sci. Rep 6:38968 27982060 10.1038/srep38968 PMC5159885

[157] Kodama M, Kodama T, Newberg JY, Katayama H, Kobayashi M, 2017. In vivo loss-of-function screens identify KPNB1 as a new druggable oncogene in epithelial ovarian cancer. PNAS 114(35):E7301–10 28811376 10.1073/pnas.1705441114 PMC5584430

[158] Wang G, Chow RD, Ye L, Guzman CD, Dai X, 2018. Mapping a functional cancer genome atlas of tumor suppressors in mouse liver using AAV-CRISPR-mediated direct in vivo screening. Sci. Adv 4(2):eaao5508 10.1126/sciadv.aao5508 PMC5829971 29503867

[159] Chow RD, Guzman CD, Wang G, Schmidt F, Youngblood MW, 2017. AAV-mediated direct in vivo CRISPR screen identifies functional suppressors in glioblastoma. Nat. Neurosci 20(10):1329–41 28805815 10.1038/nn.4620 PMC5614841

[160] Roth TL, Li PJ, Blaeschke F, Nies JF, Apathy R, 2020. Pooled knockin targeting for genome engineering of cellular immunotherapies. Cell 181(3):728–44.e21 32302591 10.1016/j.cell.2020.03.039 PMC7219528

[161] Chow RD, Chen S. 2018. Cancer CRISPR screens in vivo. Trends Cancer 4(5):349–58 29709259 10.1016/j.trecan.2018.03.002 PMC5935117

[162] Han K, Pierce SE, Li A, Spees K, Anderson GR, 2020. CRISPR screens in cancer spheroids identify 3D growth-specific vulnerabilities. Nature 580(7801):136–41 32238925 10.1038/s41586-020-2099-x PMC7368463

[163] Michels BE, Mosa MH, Streibl BI, Zhan T, Menche C, 2020. Pooled in vitro and in vivo CRISPR-Cas9 screening identifies tumor suppressors in human colon organoids. Cell Stem Cell 26(5):782–92.e7 32348727 10.1016/j.stem.2020.04.003

[164] Czerniecki SM, Cruz NM, Harder JL, Menon R, Annis J, 2018. High-throughput screening enhances kidney organoid differentiation from human pluripotent stem cells and enables automated multidimensional phenotyping. Cell Stem Cell 22(6):929–40.e4 29779890 10.1016/j.stem.2018.04.022 PMC5984728

[165] Brandenberg N, Hoehnel S, Kuttler F, Homicsko K, Ceroni C, 2020. High-throughput automated organoid culture via stem-cell aggregation in microcavity arrays. Nat. Biomed. Eng 4:863–74 32514094 10.1038/s41551-020-0565-2

[166] van de Wetering M, Francies HE, Francis JM, Bounova G, Iorio F, 2015. Prospective derivation of a living organoid biobank of colorectal cancer patients. Cell 161(4):933–45 25957691 10.1016/j.cell.2015.03.053 PMC6428276

[167] Gunasekara DB, DiSalvo M, Wang Y, Nguyen DL, Reed MI, 2018. Development of arrayed colonic organoids for screening of secretagogues associated with enterotoxins. Anal. Chem 90(3):1941–50 29281259 10.1021/acs.analchem.7b04032 PMC6028038

[168] Gracz AD, Williamson IA, Roche KC, Johnston MJ, Wang F, 2015. A high-throughput platform for stem cell niche co-cultures and downstream gene expression analysis. Nat. Cell Biol 17(3):340–49 25664616 10.1038/ncb3104 PMC4405128

[169] Verissimo CS, Overmeer RM, Ponsioen B, Drost J, Mertens S, 2016. Targeting mutant RAS in patient-derived colorectal cancer organoids by combinatorial drug screening. eLife 5:e18489 27845624 10.7554/eLife.18489 PMC5127645

[170] Finn JD, Smith AR, Patel MC, Shaw L, Youniss MR, 2018. A single administration of CRISPR/Cas9 lipid nanoparticles achieves robust and persistent in vivo genome editing. Cell Rep. 22(9):2227–35 29490262 10.1016/j.celrep.2018.02.014

[171] Mangeot PE, Risson V, Fusil F, Marnef A, Laurent E, 2019. Genome editing in primary cells and in vivo using viral-derived Nanoblades loaded with Cas9-sgRNA ribonucleoproteins. Nat. Commun 10(1):45 30604748 10.1038/s41467-018-07845-z PMC6318322

[172] Montagna C, Petris G, Casini A, Maule G, Franceschini GM, 2018. VSV-G-enveloped vesicles for traceless delivery of CRISPR-Cas9. Mol. Ther. Nucleic Acids 12:453–62 30195783 10.1016/j.omtn.2018.05.010 PMC6041463

[173] Wu J, Wu H, Nakagawa S, Gao J. 2020. Virus-derived materials: bury the hatchet with old foes. Biomater. Sci 8(4):1058–72 31697285 10.1039/c9bm01383k

[174] Rees HA, Liu DR. 2018. Base editing: precision chemistry on the genome and transcriptome of living cells. Nat. Rev. Genet 19(12):770–88 30323312 10.1038/s41576-018-0059-1 PMC6535181

[175] Komor AC, Kim YB, Packer MS, Zuris JA, Liu DR. 2016. Programmable editing of a target base in genomic DNA without double-stranded DNA cleavage. Nature 533(7603):420–24 27096365 10.1038/nature17946 PMC4873371

[176] Horlbeck MA, Gilbert LA, Villalta JE, Adamson B, Pak RA, 2016. Compact and highly active next-generation libraries for CRISPR-mediated gene repression and activation. eLife 5:e19760 27661255 10.7554/eLife.19760 PMC5094855

[177] Wilson LOW, O’Brien AR, Bauer DC. 2018. The current state and future of CRISPR-Cas9 gRNA design tools. Front. Pharmacol 9:749 30050439 10.3389/fphar.2018.00749 PMC6052051

[178] Tycko J, Wainberg M, Marinov GK, Ursu O, Hess GT, 2019. Mitigation of off-target toxicity in CRISPR-Cas9 screens for essential non-coding elements. Nat. Commun 10(1):4063 31492858 10.1038/s41467-019-11955-7 PMC6731277

[179] Doench JG, Hartenian E, Graham DB, Tothova Z, Hegde M, 2014. Rational design of highly active sgRNAs for CRISPR-Cas9-mediated gene inactivation. Nat. Biotechnol 32(12):1262–67 25184501 10.1038/nbt.3026 PMC4262738

[180] Moreno-Mateos MA, Vejnar CE, Beaudoin J-D, Fernandez JP, Mis EK, 2015. CRISPRscan: designing highly efficient sgRNAs for CRISPR-Cas9 targeting in vivo. Nat. Methods 12(10):982–88 26322839 10.1038/nmeth.3543 PMC4589495

[181] Doench JG, Fusi N, Sullender M, Hegde M, Vaimberg EW, 2016. Optimized sgRNA design to maximize activity and minimize off-target effects of CRISPR-Cas9. Nat. Biotechnol 34(2):184–91 26780180 10.1038/nbt.3437 PMC4744125

[182] Chari R, Mali P, Moosburner M, Church GM. 2015. Unraveling CRISPR-Cas9 genome engineering parameters via a library-on-library approach. Nat. Methods 12(9):823–26 26167643 10.1038/nmeth.3473 PMC5292764

[183] Tsai SQ, Zheng Z, Nguyen NT, Liebers M, Topkar VV, 2015. GUIDE-seq enables genome-wide profiling of off-target cleavage by CRISPR-Cas nucleases. Nat. Biotechnol 33(2):187–97 25513782 10.1038/nbt.3117 PMC4320685

[184] Isaac RS, Jiang F, Doudna JA, Lim WA, Narlikar GJ, Almeida R. 2016. Nucleosome breathing and remodeling constrain CRISPR-Cas9 function. eLife 5:313450 10.7554/eLife.13450 PMC4880442 27130520

[185] Horlbeck MA, Witkowsky LB, Guglielmi B, Replogle JM, Gilbert LA, 2016. Nucleosomes impede Cas9 access to DNA in vivo and in vitro. eLife 5:e12677 26987018 10.7554/eLife.12677 PMC4861601

[186] Yarrington RM, Verma S, Schwartz S, Trautman JK, Carroll D. 2018. Nucleosomes inhibit target cleavage by CRISPR-Cas9 in vivo. PNAS 115:9351–58 30201707 10.1073/pnas.1810062115 PMC6156633

[187] Zhang X-H, Tee LY, Wang X-G, Huang Q-S, Yang S-H. 2015. Off-target effects in CRISPR/Cas9-mediated genome engineering. Mol. Ther. Nucleic Acids 4:e264 26575098 10.1038/mtna.2015.37 PMC4877446

[188] Stowers RS, Shcherbina A, Israeli J, Gruber JJ, Chang J, 2019. Matrix stiffness induces a tumorigenic phenotype in mammary epithelium through changes in chromatin accessibility. Nat. Biomed. Eng 3(12):1009–19 31285581 10.1038/s41551-019-0420-5 PMC6899165

[189] Rabineau M, Flick F, Ehlinger C, Mathieu E, Duluc I, 2018. Chromatin de-condensation by switching substrate elasticity. Sci. Rep 8(1):12655 30140058 10.1038/s41598-018-31023-2 PMC6107547

[190] Makhija E, Jokhun DS, Shivashankar GV. 2016. Nuclear deformability and telomere dynamics are regulated by cell geometric constraints. PNAS 113:32–40 10.1073/pnas.1513189113 PMC4711833 26699462

[191] Toh KC, Ramdas NM, Shivashankar GV. 2015. Actin cytoskeleton differentially alters the dynamics of lamin A, HP1α and H2B core histone proteins to remodel chromatin condensation state in living cells. Integr. Biol 7(10):1309–17 10.1039/c5ib00027k 26359759

[192] Spagnol ST, Dahl KN. 2014. Active cytoskeletal force and chromatin condensation independently modulate intranuclear network fluctuations. Integr. Biol 6(5):523–31 10.1039/c3ib40226f 24619297

[193] Jokhun DS, Shang Y, Shivashankar GV. 2018. Actin dynamics couples extracellular signals to the mobility and molecular stability of telomeres. Biophys. J 115(7):1166–79 30224051 10.1016/j.bpj.2018.08.029 PMC6170704

[194] Damodaran K, Venkatachalapathy S, Alisafaei F, Radhakrishnan AV, Sharma Jokhun D, 2018. Compressive force induces reversible chromatin condensation and cell geometry-dependent transcriptional response. Mol. Biol. Cell 29(25):3039–51 30256731 10.1091/mbc.E18-04-0256 PMC6333178

[195] Chang HHY, Pannunzio NR, Adachi N, Lieber MR. 2017. Non-homologous DNA end joining and alternative pathways to double-strand break repair. Nat. Rev. Mol. Cell Biol 18(8):495–506 28512351 10.1038/nrm.2017.48 PMC7062608

[196] Chen CS, Mrksich M, Huang S, Whitesides GM, Ingber DE. 1997. Geometric control of cell life and death. Science 276(5317):1425–28 9162012 10.1126/science.276.5317.1425

[197] Irianto J, Xia Y, Pfeifer CR, Athirasala A, Ji J, 2017. DNA damage follows repair factor depletion and portends genome variation in cancer cells after pore migration. Curr. Biol 27(2):210–23 27989676 10.1016/j.cub.2016.11.049 PMC5262636

[198] Klein TJ, Glazer PM. 2010. The tumor microenvironment and DNA repair. Semin. Radiat. Oncol 20(4):282–87 20832021 10.1016/j.semradonc.2010.05.006 PMC2948843

[199] Caridi CP, D’Agostino C, Ryu T, Zapotoczny G, Delabaere L, . 2018. Nuclear F-actin and myosins drive relocalization of heterochromatic breaks. Nature 559(7712):54–60 29925946 10.1038/s41586-018-0242-8 PMC6051730

[200] Le HQ, Ghatak S, Yeung C-YC, Tellkamp F, Günschmann C, 2016. Mechanical regulation of transcription controls Polycomb-mediated gene silencing during lineage commitment. Nat. Cell Biol 18(8):864–75 27398909 10.1038/ncb3387

[201] Caiazzo M, Okawa Y, Ranga A, Piersigilli A, Tabata Y, Lutolf MP. 2016. Defined three-dimensional microenvironments boost induction of pluripotency. Nat. Mater 15(3):344–52 26752655 10.1038/nmat4536

[202] Kim E, Tae G. 2016. Direct reprogramming and biomaterials for controlling cell fate. Biomater. Res 20:39 27980804 10.1186/s40824-016-0086-y PMC5142385

[203] Kulangara K, Adler AF, Wang H, Chellappan M, Hammett E, 2014. The effect of substrate topography on direct reprogramming of fibroblasts to induced neurons. Biomaterials 35(20):5327–36 24709523 10.1016/j.biomaterials.2014.03.034 PMC4023960

[204] Smith DK, Yang J, Liu M-L, Zhang C-L. 2016. Small molecules modulate chromatin accessibility to promote NEUROG2-mediated fibroblast-to-neuron reprogramming. Stem Cell Rep. 7(5):955–69 10.1016/j.stemcr.2016.09.013 PMC5106529 28157484

[205] Crowder SW, Leonardo V, Whittaker T, Papathanasiou P, Stevens MM. 2016. Material cues as potent regulators of epigenetics and stem cell function. Cell Stem Cell 18(1):39–52 26748755 10.1016/j.stem.2015.12.012 PMC5409508

[206] Li Y, Chu JS, Kurpinski K, Li X, Bautista DM, 2011. Biophysical regulation of histone acetylation in mesenchymal stem cells. Biophys. J 100(8):1902–9 21504726 10.1016/j.bpj.2011.03.008 PMC3077706

[207] Downing TL, Soto J, Morez C, Houssin T, Fritz A, 2013. Biophysical regulation of epigenetic state and cell reprogramming. Nat. Mater 12(12):1154–62 24141451 10.1038/nmat3777 PMC9675045

[208] Vining KH, Mooney DJ. 2017. Mechanical forces direct stem cell behaviour in development and regeneration. Nat. Rev. Mol. Cell Biol 18(12):728–42 29115301 10.1038/nrm.2017.108 PMC5803560

[209] Miroshnikova YA, Nava MM, Wickström SA. 2017. Emerging roles of mechanical forces in chromatin regulation. J. Cell. Sci 130(14):2243–50 28646093 10.1242/jcs.202192

[210] Uhler C, Shivashankar GV. 2017. Regulation of genome organization and gene expression by nuclear mechanotransduction. Nat. Rev. Mol. Cell Biol 18(12):717–27 29044247 10.1038/nrm.2017.101

[211] Chang L, Azzolin L, Di Biagio D, Zanconato F, Battilana G, 2018. The SWI/SNF complex is a mechanoregulated inhibitor of YAP and TAZ. Nature 563(7730):265–69 30401838 10.1038/s41586-018-0658-1 PMC7612964

[212] Jain N, Iyer KV, Kumar A, Shivashankar GV. 2013. Cell geometric constraints induce modular gene-expression patterns via redistribution of HDAC3 regulated by actomyosin contractility. PNAS 110(28):11349–54 23798429 10.1073/pnas.1300801110 PMC3710882

[213] Tajik A, Zhang Y, Wei F, Sun J, Jia Q, 2016. Transcription upregulation via force-induced direct stretching of chromatin. Nat. Mater 15(12):1287–96 27548707 10.1038/nmat4729 PMC5121013

